# Exploring the In situ pairing of human galectins toward synthetic *O*-mannosylated core M1 glycopeptides of α-dystroglycan

**DOI:** 10.1038/s41598-022-22758-0

**Published:** 2022-10-23

**Authors:** Lareno L. Villones, Anna-Kristin Ludwig, Hiroyuki Kumeta, Seiya Kikuchi, Rika Ochi, Tomoyasu Aizawa, Shin-Ichiro Nishimura, Hans-Joachim Gabius, Hiroshi Hinou

**Affiliations:** 1 Frontier Research Center for Advanced Material and Life Science, Graduate School of Life Science and Faculty of Advanced Life Science, Hokkaido University, N21, W11, Sapporo, 001-0021 Japan; 2 Physiological Chemistry, Department of Veterinary Sciences, Faculty of Veterinary Medicine, Ludwig-Maximilians-University Munich, 82152 Planegg-Martinsried, Germany

**Keywords:** Biophysics, Chemical biology, Structural biology, Chemical biology, NMR spectroscopy, Carbohydrate chemistry

## Abstract

Dystroglycan (DG), which constitutes a part of the dystrophin–glycoprotein complex, connects the extracellular matrix to the cytoskeleton. The matriglycans presented by the extracellular α-DG serve as a contact point with extracellular matrix proteins (ECM) containing laminin G-like domains, providing cellular stability. However, it remains unknown whether core M1 (GlcNAcβ1-2Man) structures can serve as ligands among the various *O*-Mannosylated glycans. Therefore, based on the presence of *N*-acetylLactosamine (LacNAc) in this glycan following the core extension, the binding interactions with adhesion/growth-regulatory galectins were explored. To elucidate this process, the interaction between galectin (Gal)-1, -3, -4 and -9 with α-DG fragment ^372^TRGAIIQTPTLGPIQPTRV^390^ core M1-based glycopeptide library were profiled, using glycan microarray and nuclear magnetic resonance studies. The binding of galectins was revealed irrespective of its modular architecture, adding galectins to the list of possible binding partners of α-DG core M1 glycoconjugates by *cis*-binding (via peptide- and carbohydrate-protein interactions), which can be abrogated by α2,3-sialylation of the LacNAc units. The LacNAc-terminated α-DG glycopeptide interact simultaneously with both the S- and F-faces of Gal-1, thereby inducing oligomerization. Furthermore, Gal-1 can trans-bridge α-DG core M1 structures and laminins, which proposed a possible mechanism by which Gal-1 ameliorates muscular dystrophies; however, this proposal warrants further investigation.

## Introduction

**Figure 1:**
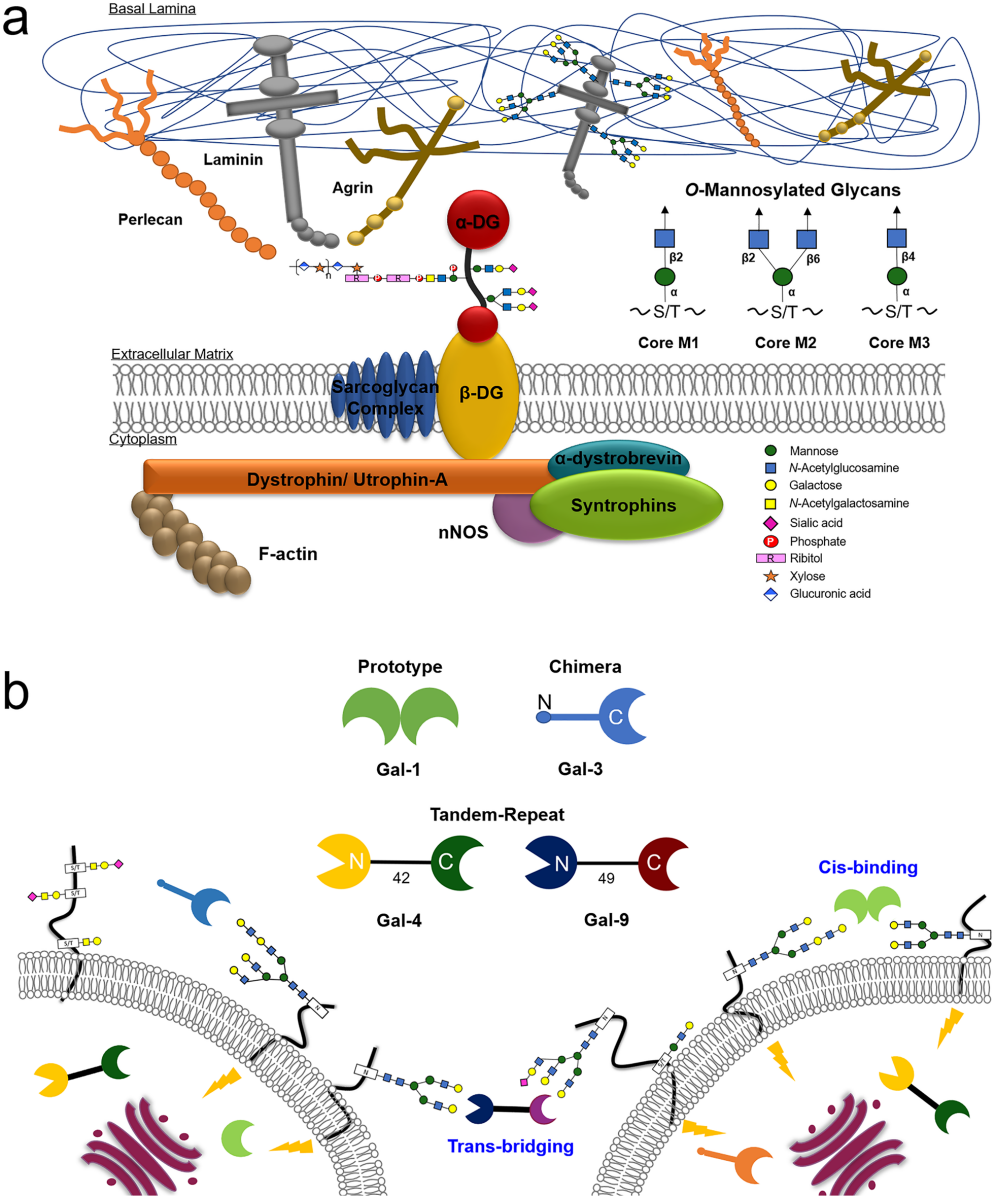
The dystrophin-glycoprotein complex (DGC) and *O*-mannosylated glycans identified on α-DG (**a**). Galectin test panel covering the three types of vertebrate galectins: prototype (Gal-1), chimera (Gal-3), and tandem-repeat type (Gal-4 and -9) (**b**). CRD positioning relative to the termini is designated by N and C. The cis-binding and trans-bridging activity of galectin is also illustrated.

Protein *O*-mannosylation is an evolutionarily conserved modification observed in fungi and metazoans. In animals, the *O*-linked mannose (*O*-Man) exists in a limited number of proteins that are required for normal development and have vital functions in muscle and neural physiology^1,2^. The α-dystroglycan (α-DG) is the extracellular component of dystroglycan (DG) (Fig. 1a), and is the most extensively studied mammalian *O*-Man glycoprotein. It is ubiquitously expressed in the skeletal muscles and the brain and is associated with cell adhesion, muscle integrity, and neurological development^1–3^. α-DG also acts as a receptor for pathogens such as *Mycobacterium leprae* and arenaviruses^4,5^.

α-DG possesses unique glycans, i.e. NeuAcα2-3Galβ1-4GlcNAcβ1-2Manα1-*O*-Ser/Thr, in its mucin (MUC)–like domain (amino acid residues 317–488)^6^. Subsequent studies have identified three types of *O*-Man glycan structures and have classified them based on the linkage of GlcNAc to Man^1^: core M1 (GlcNAcβ1-2Manα1-*O*-Ser/Thr); core M2 (GlcNAcβ1-6(GlcNAcβ1-2)Manα1-*O*-Ser/Thr); and core M3 (GlcNAcβ1-4Manα1-*O*-Ser/Thr), as shown in Fig. 1a. The DG in skeletal muscle is intimately involved in cell–extracellular matrix (-ECM) communication via the interaction between the α-DG extended core M3 (matriglycan) and the extracellular carbohydrate-binding proteins (CBPs) that contain laminin G-like (LG) domains, such as laminins^7^. This association facilitates the interaction of the β-DG with various cytoplasmic proteins, such as dystophin, and ultimately with the F-actin, establishing the extra- and intracellular connections. This DG-LG relay system mediates the assembly of the basement membrane, providing muscular stability. Although the functional role of core M3 has been extensively studied, the (patho)physiological roles of the core M1-related structures are relatively less investigated.

In α-DG, about half of *O*-glycans are *O-*mannosylated^8^. Interestingly, proper expression of the core M1 and M2 glycans (accounting for 15% and ~ 5% *O*-Man type glycans in mammalian brain, respectively) is necessary for functional core M3 extension of the α-DG^9,10^. The complete loss matriglycan structure owing to mutations in protein *O*-linked mannose β1,2N-acetylglucosaminyltransferase-1 (POMGnT1), fukutin (FKTN), and fukutin-related protein (FKRP) leads to a group of congenital muscular dystrophy referred to as α-dystroglycanopathy, a neuromuscular disorder which is characterized by progressive muscular degeneration, inaccurate brain formation, intellectual disability, and ocular anomalies^1,11,12^. These aberrations are currently intractable and various therapeutic approaches have been actively explored^13^. Intriguingly, a complete loss of MUC-type core 2 *O*-glycans and branched core 1 antennae has been found to lead to a possible functional compensatory upregulation of the *O*-Man glycans in the stomach of engineered mice^14^. Since the mentioned MUC-type *O*-glycans are docking sites for tissue lectins underlying a broad range of (patho)physiological activities^15^, it is interesting to explore the capacity of *O*-Man glycans to interact with the human lectins, such as the adhesion/growth-regulatory galectins.

Galectins share a β-sandwich-type carbohydrate recognition domain (CRD) that specifically binds to β-galactosides and pair with distinct counterreceptors to trigger a broad range of post-binding events, such as cell growth/adhesion/differentiation, immune response, inflammatory functions, and tumor development and progression^16,17^. Based on the structural aspects of human galectins, three designs can occur in nature (Fig. 1b). Each member of this endogenous lectin family (“readers”) decipher and translate glycan messages depending on its modular architecture as well as the glycan structure and the modification on the galactose residues (“sugar code”)^18^. Galectin (Gal)-1, -3, -4, and -9 are expressed by the muscle and neural cells exhibiting critical, yet conflicting roles that remain elusive^19–23^. These galectin interactions are physiologically relevant mainly through cis-/trans-bridging (Fig. 1b) of the cell surface-associated components in the skeletal muscles and nervous system^20,24,25^.

Evidently, the presence of glycan chains on the cellular glycoproteins is functionally relevant when pairing with tissue lectins. This phenomenon has aroused an interest in exploratory studies involving synthetic glycopeptides and lectin panels. Our recent work using chemoenzymatic synthesis and microarray technology has demonstrated that the core M1 extended mucin-like domain of the α-DG fragment, i.e. ^372^TRGAIIQ**T**P**T**LGPIQP**T**RV^390^, interacts with various plant lectins but not with laminins^26^. Using this experimental set-up, glycopeptide microarray technology has been previously shown to be a robust tool for determining the selective pairing of galectins with their binding partners in the MUC1-based glycopeptide library^27^. In line with previous works, this study investigated the interaction profile of galectins with the core M1 glycan-bearing glycopeptides. First, the α-DG core M1 glycoconjugate library was synthesized and their binding to the plant lectins was used as internal controls. The interactions of the *O*-Man core M1 ligands with the galectin test panel were initially profiled using microarray. The nuclear magnetic resonance (NMR) experiments with Gal-1 further validated the affinity of galectin to the prepared glycoconjugates. Overall, our findings revealed galectins can recognize the α-DG peptide and LacNAc-terminated core M1 α-DG glycopeptide, which was diminished by α2,3-sialylation of the LacNAc units. Furthermore, here we demonstrated the capability of Gal-1 to trans-bridge core M1 α-DG glycoconjugates with various laminins in microarray, providing an additional insight on the therapeutic application of this galectin in muscular dystrophy.

## Results and discussion

### Core M1-based α-DG glycopeptide microarray and quality control

**Figure 2:**
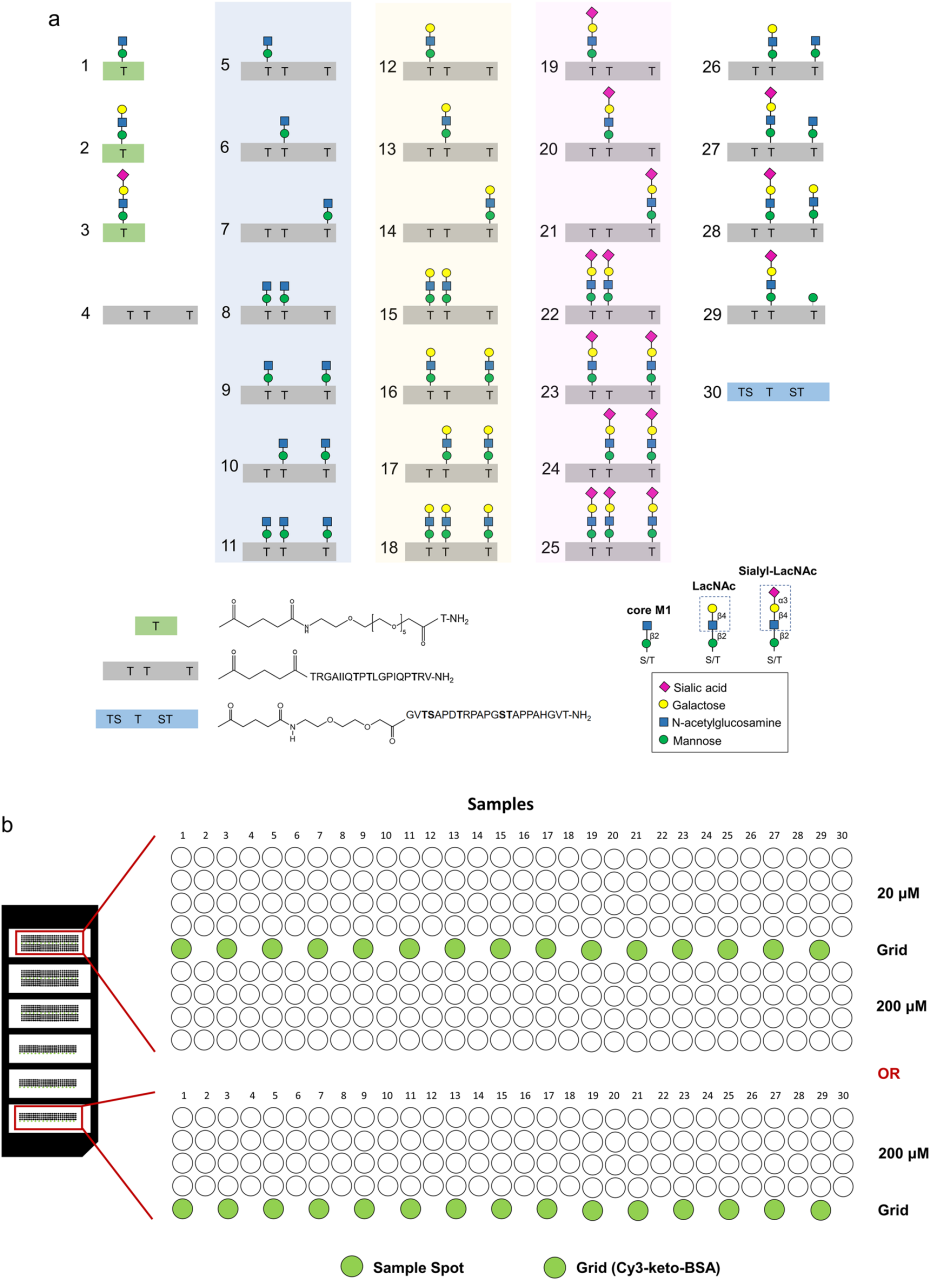
Glycoblotting-based microarray for galectin interaction profiling with core M1 modification. Synthetic α-DG core M1 glycoconjugates used for microarray experiment (**a**). Compounds were robotically printed in quadruplets at 20 and/or 200 μM on an aminooxy- coated plastic slide. Subsequently, a six-chamber rubber silicon sheet was attached. Green spots correspond to cyanine 3-keto-BSA (Cy3-keto-BSA) as grid (**b**).

Our library consisted of 30 compounds comprising the controls and the test glycopeptides as follows: core M1-related glyco-amino acids **1**–**3**, α-DG peptide backbone **4** (TRGAIIQTPTLGPIQPTRV), glycopeptides **5**–**29** presenting the core M1-related oligosaccharides at potential *O*-mannosylation sites of α-DG (Thr379, Thr381, and Thr388), and MUC1 peptide **30** (GVTSAPDTRPAPGSTAPPAHGVT) as an additional peptide control (Fig. 2a and Supplemental Table S1). Glyco-amino acid **1**, nonglycosylated peptides **4** and **30**, and glycopeptides **5**–**11** with systematically arranged mono-, bis-, and tris-core M1 were synthesized by the standard microwave-assisted solid-phase glycopeptide synthesis terminated with reactive ketone linker at the *N*-terminus^28^. The core M1 disaccharide of compounds **1** and **5**–**11** was extended by adding a galactose unit using β1,4-galactosyltransferase and UDP-galactose for generating compounds **2** and **12**–**18**, respectively. These compounds were further elongated by the addition of sialic acid with α2,3-sialyltransferase and CMP-*N*-acetylneuraminic acid, producing compounds **3** and **19**–**25**. Glycopeptides **26**–**29** displaying heterogeneous core M1 structures along α-DG peptide scaffold were also prepared by a chemoenzymatic protocol. Subsequently, 20 or 200 μM solutions of compounds **1**–**30** were used for compound printing onto the surface of a hydroxylamine-functionalized microarray chip in quadruplets, in a six-chamber format (Fig. 2b) covalently attached via an oxime bond^29^. For array-based screening with α-DG glycopeptides, the positive and negative control data using plant lectins were presented, before measuring the interaction profiles with a galectin test panel.

Five legume lectins were probed for their accessibility toward cognate glycans, especially mannose, using *Concanavalia ensiformis* agglutinin (ConA) (Supplemental Fig. S1). This positive control worked satisfactorily, and the negative controls *Dolichos biflorus* agglutinin (DBA) and *Ulex europaeus* agglutinin I (UEA I) did not generate any carbohydrate-dependent signal, as expected. Galactose or sialic acid were selectively sensed as a minor ligand [*Glycine max* (soybean) agglutinin, SBA or *Triticum vulgaris* (wheat-germ) agglutinin, WGA, respectively] yielded signals at appropriate positions. These results confirmed the validity of the protocol for monitoring the affinity of each member of the galectin panel toward the prepared glycoconjugate library.

### Galectins evaluated

Our galectin test panel included three types of architectural design found in humans: the non-covalently-associated homodimeric CRD (prototype) Gal-1; the chimera-type Gal-3 that has a single CRD and a non-lectin *N*-terminal, inducing self-association forming a pentamer; and the linker-connected heterodimeric CRD with different glycan-binding affinities (tandem-repeat type) Gal-4 and -9 (Fig. 1b). These human galectins share binding preference to Lac/LacNAc, with variability for other types of natural glycans such as LacdiNAc (GalNAcβ1-4GlcNAc) or 3’-*O*-sulfated galactose, as revealed by systematic frontal affinity chromatography studies^30^ or aggregation assays with surface-programmable nanoparticles^31^.

Among the test galectins, Gal-1 is unique because it contains six cysteine residues making it highly sensitive to oxidation resulting in decreased lectin binding activity^32,33^. However, under non-reducing conditions, Gal-1, but not Gal-3, promotes nerve growth and axonal regeneration at a low concentration (50 pg/mL)^34^. Moreover, 10 μg/mL Gal-1, -3, and -9 (except for Gal-4) presented almost the same interaction with Galβ1,4GlcNAcβ1,2Manα (LacNAc core M1 glycan) with a thiol linker immobilized in a maleimide-functionalized microarray^35^. Therefore, the galectins were tested in a screening concentration range of 0.10 to 32.0 μg/mL in the absence of any reducing agents and used ambient air (partially oxidizing conditions). Since half of the *O*-glycans present in α-DG are mucin-type glycans found in MUC1^8^, before testing the whole core M1 α-DG glycopeptide library, we first surveyed the affinity of galectins to selected galactose-terminated MUC1 and α-DG glycoconjugates under the fixed microarray conditions (Supplemental Fig. S2).

### Galactose-terminated MUC1 versus α-DG core M1 *O*-glycans

The positive controls ConA and PNA (galactose-binding lectin) yielded signals that effectively distinguished between *O*-Man and MUC1 glycoconjugates^26,36^ (Supplemental Fig. S3). Notably, only *O*-Man glycopeptides showed interactions at the tested galectin concentrations. The highly expressed short determinants of MUC1 glycoprotein in cancer, such as core 1 (TF antigen, Galβ1,3GalNAcα1-*O*-Ser/Thr) and core 2 (Galβ1-3[GlcNAcβ1-6]GalNAcα1-*O*-Ser/Thr), were identified as binding receptors for Gal-3, -4, and -8 at 90.0 µg/mL concentration, with no interaction with Gal-1 even at high lectin concentrations^27^. Furthermore, a strong interaction of MUC1-bearing TF antigen with Gal-3 is well documented at high concentrations, but not with Gal-1^37,38^. This suggests that the tested galectins bind to the *O-*Man ligands with higher affinity than the mucin type *O*-glycans in the array. Thus, the complete screening of the prepared core M1 α-DG glycopeptide library against galectins is beneficial and of interest.

### Galectin binding profile with α-DG core M1 glycoconjugates.

**Figure 3:**
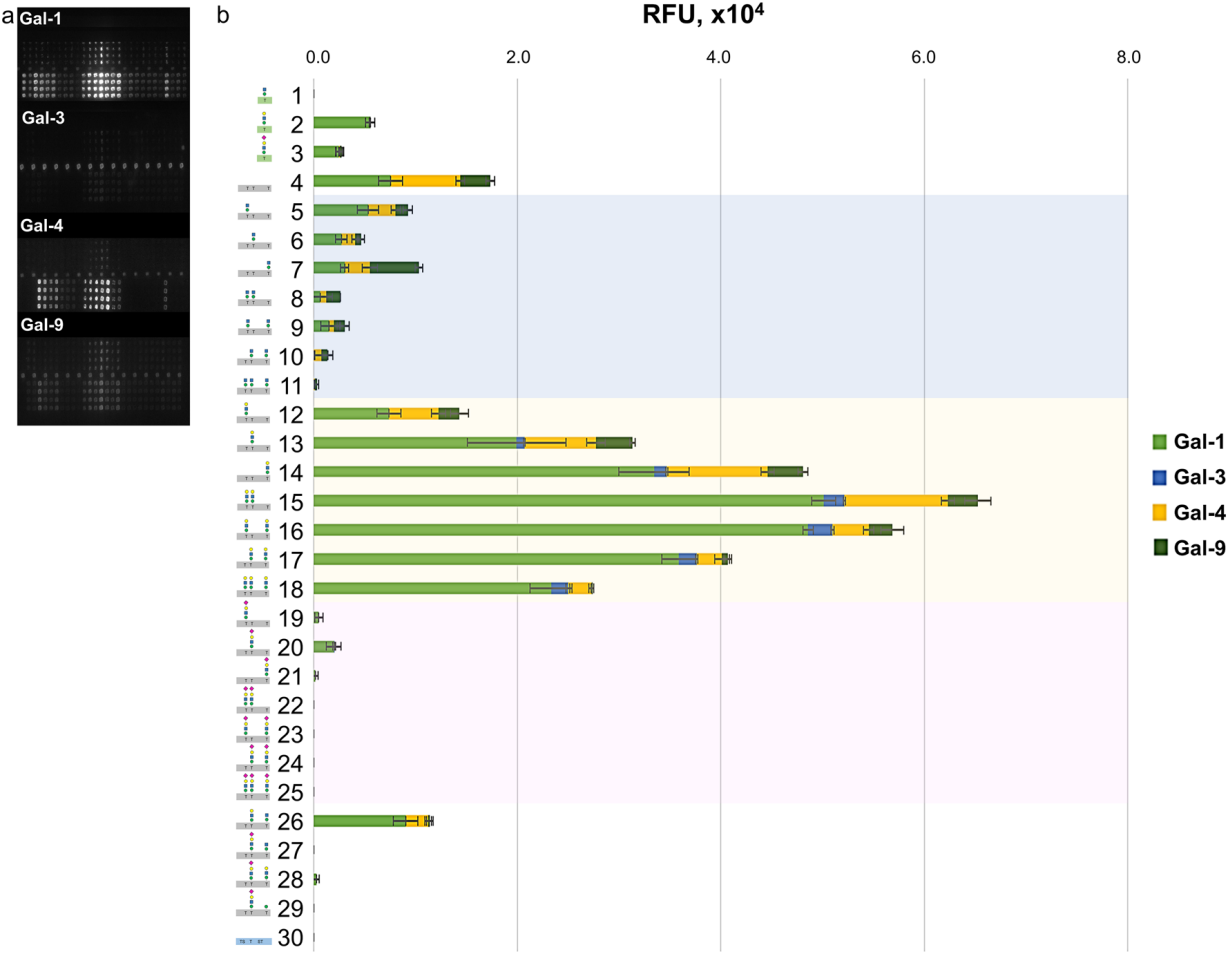
Fluorescence image of microarray chip is taken after treatment of 20 and 200 μM core M1 α-DG glycoconjugates with 10.0 μg/mL galectin solution (**a**). Stacked chart of signal intensities of 200 μM core M1 α-DG glycoconjugates with 3.20 μg/mL of wild-type galectins (**b**). The relative interaction of 20 and 200 μM core m1 α-DG glycoconjugates with 0.10 μg/mL to 32.0 μg/mL Gal-1, -3, -4, and -9 are in Supplementary Fig. S4 and S5, respectively.

In a partially oxidizing ambient air environment, the prototype Gal-1 could bind to the glycans of the test panel such that strong signals were recorded (Fig. 3a,b and Supplemental Figs. S4a,b and S5a,b). Gal-1 failed to bind to the core M1 glyco-amino acid **1**, whereas β1,4-galactose extension (**2**) led to an enhanced affinity. Further elongation of the core M1 structure with α2,3-NeuAc (**3**) resulted in minimal signal intensity reduction. Notably, binding was observed with unglycosylated peptide **4** of α-DG, but not with MUC1 peptide **30**. In contrast, the core M1 attached to the α-DG peptide backbone (**5**–**11**) exhibited binding activity to Gal-1. There was a notable decrease in binding interaction occurred when multiple core M1 disaccharide units were present along the peptide sequence (**5**–**7** vs. **8**–**10** vs. **11**). The wild-type protein bound to the LacNAc-terminated core M1 structures (**12**–**18**, **26**, and **28**). In complex-type biantennary *N*-glycans, Gal-1 has a high affinity for α2,3-sialylated poly-LacNAc, while the binding is completely restricted by α2,6-sialylation^39,40^. However, α2,3-sialylation at the LacNAc-terminated core M1 diminished Gal-1 binding to β-galactoside in *O*-Man glycans (**12**–**18** vs. **19**–**25**), and even at a single glycosylation site (**17** vs. **28**). This difference in Gal-1 affinity towards α2,3-sialylated *N*-linked and *O*-Man glycans is attributed to the conformational change induced by the presence of other carbohydrate moieties along the glycan structure and the type of linkage to the protein^26,41,42^. Given the redox sensitivity of Gal-1, the binding interaction with α-DG glycoconjugates in a reducing environment containing DTT was verified (Supplemental Fig. S6). Under reducing conditions, Gal-1 presented a similar binding behavior but failed to bind to the core M1-terminated **5**–**11**, differentiating Gal-1 activity in the absence of DTT.

The chimera-type Gal-3 showed a weak affinity for the LacNAc-terminated glycopeptides of α-DG (**12**–**18**) (Fig. 3a,b and Supplemental Figs. S4a,c and S5a,c). This observation was in agreement a recent report where Gal-3 have less interactions with the terminal LacNAc epitopes^43^. The position of the LacNAc unit along the peptide sequence (**12** vs. **13** vs. **14**) affected Gal-3 binding. Bis-LacNAc bearing **15** demonstrated stronger affinity than mono-LacNAc **12**–**14**, because of multivalency at consecutive glycosylation sites. However, longer distance between the two LacNAc units led to reduced interaction (**15** vs. **16**–**17**). Furthermore, the presence of more than two LacNAc units (**18**) did not result in higher intensity. Similar to Gal-1, these interactions were completely abrogated by the α2,3-sialic acid extension of the LacNAc units (**19**–**25**).

The tandem-repeat types Gal-4 and -9 presented similar binding patterns but with different signal intensities to α-DG glycoconjugates. Both galectins interacted with peptide **4,** mono-core M1 substituted (**5**–**7**), and LacNAc-terminated core M1 glycopeptides (**12**–**18**) (Fig. 3a,b and Supplemental Figs. S4a,d,e and S5a,d,e). The influence of LacNAc position and density affected the binding of these lectins as previously observed with Gal-1 and -3. Overall, Gal-4 showed a higher interaction at low concentrations than Gal-9. In contrast, only Gal-9 revealed interactions with glycol-amino acids (**2** and **3**) and α2,3-Sia terminated glycopeptides (**19**–**25**, **27**–**29**), thus distinguishing its binding activity with Gal-4. Likewise, α2,3-sialylation of the LacNAc units resulted in a significant reduction in the affinity even with **28** (in the case of Gal-4), which displayed a single LacNAc terminal on one of the glycosylation sites, implying that the different glycosylation of neighboring amino acids can disrupt the affinity of the CBPs.

The glycan-CBP interactions are influenced by not only the type of glycan but also the spatial orientation, carrier scaffold, accessibility, density, and spacing of sugar moieties along the glycoconjugate, to obtain optimum fit within the binding pocket of the receptors^37,40,44^. In a previous report, Gal-1, -3, and -9, but not Gal-4, revealed similar affinity to the LacNAc- terminated *O*-Man glycan in a microarray^35^. In this study, the addition of Thr to the reducing end of LacNAc-terminated *O*-Man affected the binding interactions of galectins (Gal-1>>Gal-4≈Gal-9, but not Gal-3, Fig. 3 and Supplemental Figs. S4 and S5). When this LacNAc-terminated core M1 glyco-amino acid structure (**2**) was presented to the α-DG peptide scaffold (**12**–**14**), the binding affinities of the tested galectins were significantly enhanced. This effect was also observed for the TF antigen–Gal-3 interaction (K_d_ = 245 µM), which was significantly enhanced when the TF antigen was displayed along the MUC1 peptide scaffold (K_d_ = 45 µM)^37^. Furthermore, bis-LacNAc presenting α-DG glycopeptides demonstrated the highest signal intensity (**15**–**17**). The tris-LacNAc terminated α-DG glycopeptide (**18**) showed lesser interaction due to the close proximity of the sugar components along the peptide scaffold resulting in galectin steric hindrance.

### Validation of the galectin interaction

**Figure 4:**
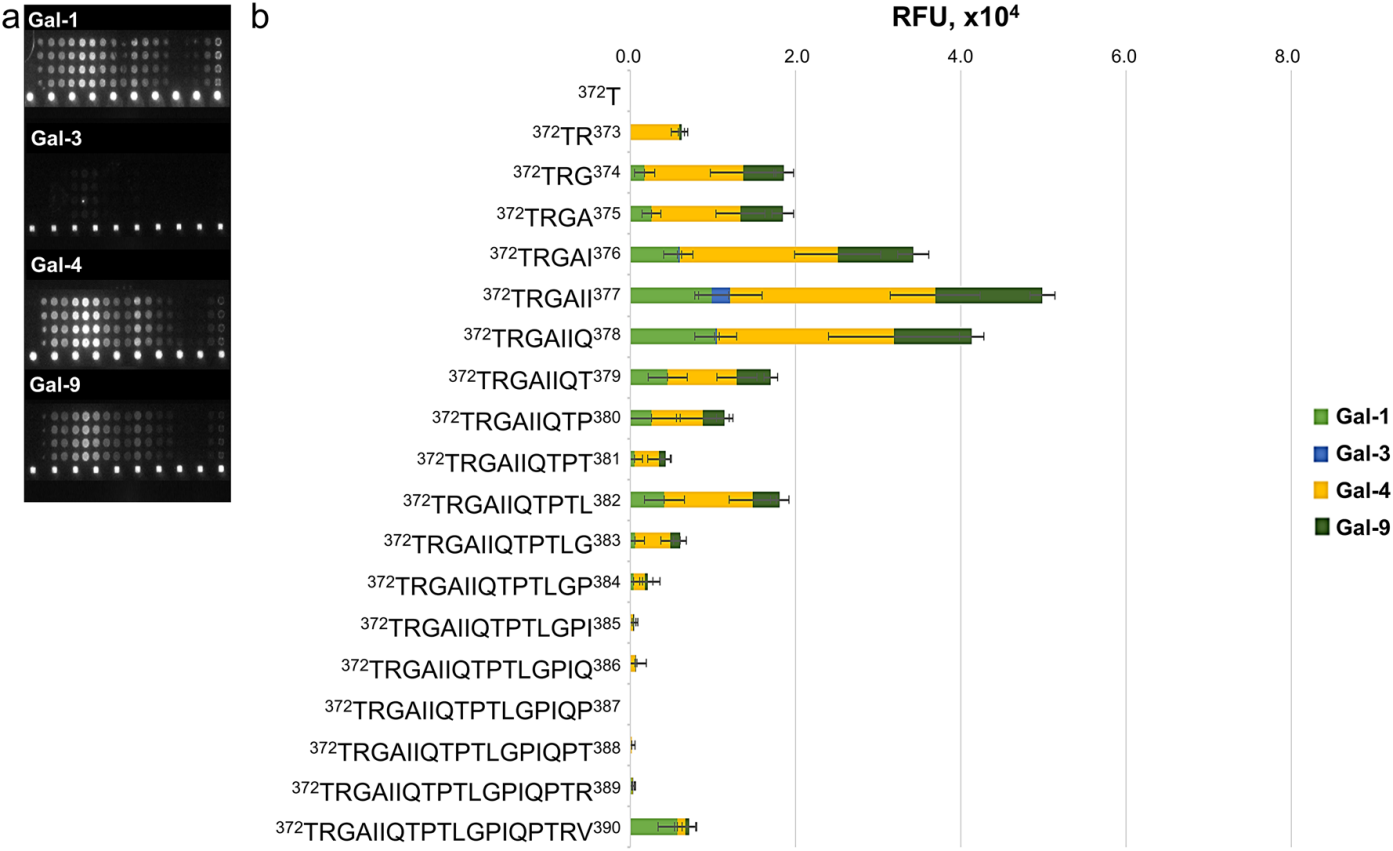
Fluorescence image of microarray chip of α-DG unglycosylated peptide library taken after treatment with 10.0 μg/mL galectin solution (**a**). Stacked chart of signal intensities of 200 μM unglycosylated peptide of α-DG library with 3.20 μg/mL Gal-1, -3, -4, and -9 (**b**). The relative interaction of 200 μM α-DG unglycosylated peptides with 0.10 μg/mL to 32.0 μg/mL Gal-1, -3, -4, and -9 is in Supplementary Fig. S7. p = Peptide.

Gal-1, -4 and -9 (except -3) were found to interact with peptide **4** of α-DG, but failed to bind with peptide **30** of MUC1, demonstrating the specificity of these galectins towards certain peptide sequences. Microarray peptide epitope mapping experiments suggested that the presence of hydrophobic amino acids at position ^374^GAII^377^ is responsible for the binding of unglycosylated peptide **4** to galectins (Fig. 4 Supplemental Figs. S7), indicating that hydrophobic interactions play an important role. This is analogous to the binding of Gal-1 to peptide ligands λ5-UR22-45 (52mer, α-helix) and Anginex (33mer, β-sheet)^45,46^. The unglycosylated peptide **4** (19mer, random coil) displays no particular fold similar to the full-length α-DG peptide and needs to be glycosylated to attain a stable structure^47^. The presence of core M1 at positions Thr379, Thr381, and Thr388 of the α-DG peptide backbone (**5**–**7**) lowered the binding affinity. Furthermore, the presence of the bis- and tris-core M1 structures (**8**–**11**) resulted in a remarkable decrease in the interaction profiles. This can be accounted for by the conformational changes documented in the peptide backbone caused by additional core M1 units along the scaffold, from a random coil-like structure to a turn-like conformation^26^. This is analogous to our observation on the effect of MUC1 peptide glycosylation with multiple Tn antigen (GalNAcα1-Ser/Thr) and its interaction with macrophage galactose lectin^48,49^. Therefore, the glycans attached to the peptide scaffold evidently influence the entropic properties of the peptide backbone by changing the secondary structure^38^, thereby affecting the lectin affinity. The galectins revealed high interaction with LacNAc-terminated glycoconjugates (**2**, **12**–**18**, **26**), further extension of the LacNAc units with α2,3-sialic acid led to a reduction in the galectin affinity, as demonstrated by **3**, **19**–**25** and **27**–**29**. To verify whether these observed interactions were dependent on the activity of galectin CRD, the influence of sugar inhibitor and *N*-acetylated peptide **4** was determined.

**Figure 5:**
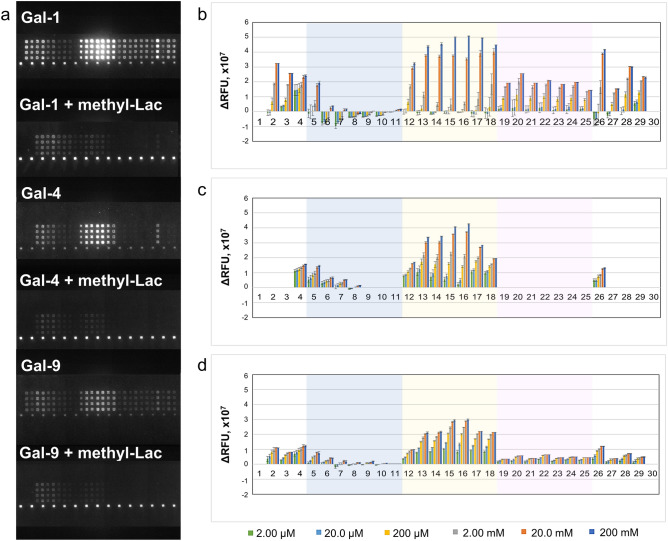
Fluorescence image of microarray chip is taken after treatment of 10.0 μg/mL lectin solution with 200 mM methyl-Lac (**a**) and relative binding properties of 200 μM core M1 αDG glycoconjugates with Gal-1 (**b**), Gal-4 (**c**), and Gal-9 (**d**) with the presence of 2.00 µM to 200 mM methyl-Lac. ΔRFU = RFU_control (no inhibitor) _− RFU_methyl-lac (or other inhibitor)_.

Galectin CRD comprises eleven antiparallel β-strands: β1, β10, β3-6 (S-face; canonical sugar-binding site) and β11, β2, β7-9 (F-face)^17^. The presence of methyl-β-lactoside (methyl-Lac), a natural ligand of the S-face, resulted in a significantly reduced interaction of galectins with the glycopeptides, specifically the LacNAc-terminated glycoconjugates **2**, **12**–**18**, and **26** (Fig. 5a–d). Moreover, the addition of non-competing osmolarity controls (maltose and cellobiose) and *N-*acetylated peptide **4** had minimal effect on the binding (Supplemental Figs. S8–S10). Thus, these binding activities depend on the S-face of the galectins. Intriguingly, the binding to the compounds **4**–**7** was not completely inhibited, when the S-face was blocked by excess (200 mM) methyl-Lac, proposing that the peptide region of α-DG plays a role in the binding event.

### Interaction between Gal-1 and core M1 glycopeptides by NMR

**Figure 6:**
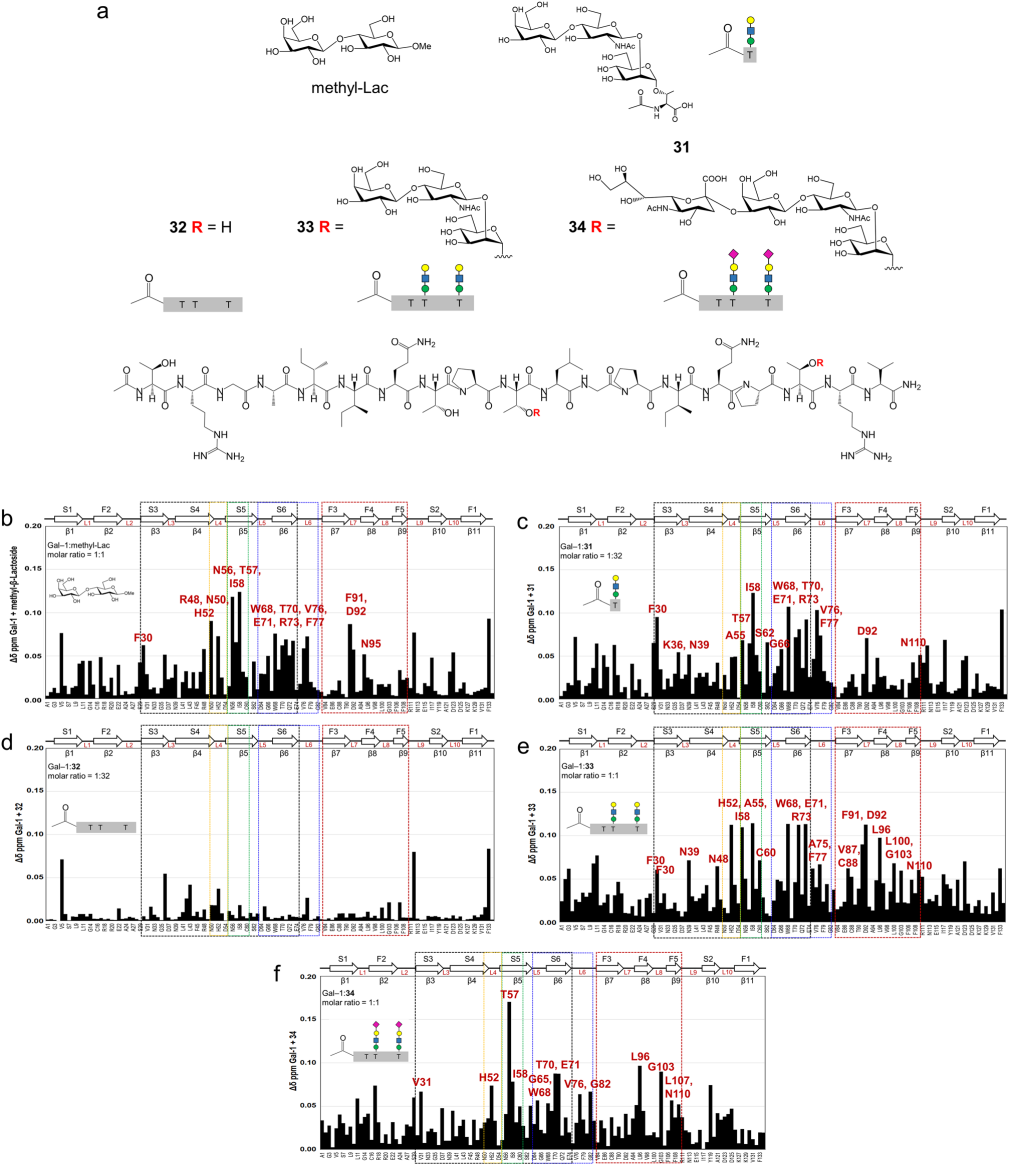
Structure of methyl-Lac, **31**, **32**, **33**, and **34** used for ^1^H-^15^N HSQC NMR studies (**a**). Chemical shift map (Δδ vs. sequence of Gal-1) is shown for methyl-Lac (**b)**, glyco-amino acid **31** (**c**), unglycosylated α-DG glycopeptide **32** (**d**), LacNAc-terminated glycopeptide **33** (**e**), sialyl-LacNAc-terminated glycopeptide **34** (**f**). Chemical shift differences (Δδ) were calculated as [(Δ^1^H)^2^ + (0.25Δ^15^N)^2^)]^1/2^. Solution condition were 20 mM potassium phosphate buffer, pH 6.9, 50 μM EDTA, with 10 mM DTT made up using a H_2_O/D_2_O (95:5%).

To verify the binding modes of Gal-1 with core M1 α-DG glycopeptides, Gal-1 NMR experiments were performed with acetylated *N*-termini compounds **31**, **32**, **33**, and **34** (Fig. 6a), corresponding to **2**, **4**, **17**, and **24**, respectively. For screening, ^15^N-labeled Gal-1 was prepared and tested under oxidizing and reducing conditions. The Gal-1 ^1^H-^15^N heteronuclear single quantum coherence (HSQC) experiments performed under oxidizing conditions (air oxidation or addition of oxidizing agents; CuSO_4_ or tetramethylazodicarboxamide) resulted in substantial aggregation of Gal-1^50^ with undetectable ^1^H-^15^N HSQC signals (Supplemental Fig. S11). In contrast, Gal-1 produced assignable signals under reducing conditions in the presence of DTT, as previously reported^51^. The chemical shift perturbation of the ^15^N-labeled Gal-1 under reducing conditions was analyzed with increasing concentration of methyl-Lac (positive control), **31**, **32**, **33**, and **34**.

The presence of methyl-Lac shifted the ^1^H-^15^N HSQC signals corresponding to the residues on S3–L6 regions and induced a minor effect on the L7–F4 regions of Gal-1, similar to lactose in a concentration-dependent manner (Fig. 6b and Supplemental Fig. S12a)^52^. High concentration of LacNAc-terminated glyco-amino acid **31** induced remarkable perturbation of the chemical shift at the S-face of Gal-1 (molar ratio 1:32), similar to that of methyl-Lac (molar ratio 1:1) (Fig. 6c and Supplemental Fig. S12b). In contrast, unglycosylated peptide **32** failed to induce any significant deviation in the chemical shift at the tested concentrations (Fig. 6d and Supplemental Fig. S12c). Low concentration of LacNAc-terminated glycopeptide **33** (molar ratio of 1:1) resulted in alterations of the chemical shifts at S-face (S4–S6 loop (L6)) and F-face (F3–F5) (Fig. 6e and Supplemental Fig. S122d). Similar regions in Gal-1 were shifted by sialyl-LacNAc-terminated glycopeptide **34** (molar ratio of 1:1); however, reduced deviations were observed on S4 and F3 regions (Fig. 6f and Supplemental Fig. S12e). At a molar ratio greater than 1:1 for both **33** and **34**, a significant reduction of Gal-1 signal intensities were observed (Supplemental Fig. S12d,e). This was attributed to Gal-1 oligomerization via cross-linking due to LacNAc binding to the S-face, and the hydrophobic binding of the α-DG peptide scaffold and the F-face. These supramolecular lectin-ligand complexes were undetectable by NMR, similar to glyco-amino acid **31** at high concentration (1:64) (Supplemental Fig. S12b). This phenomenon was also evident on Gal-1 binding with several multi-LacNAc-containing ligands such as galactorhamnogalacturonate^53^.

**Figure 7:**
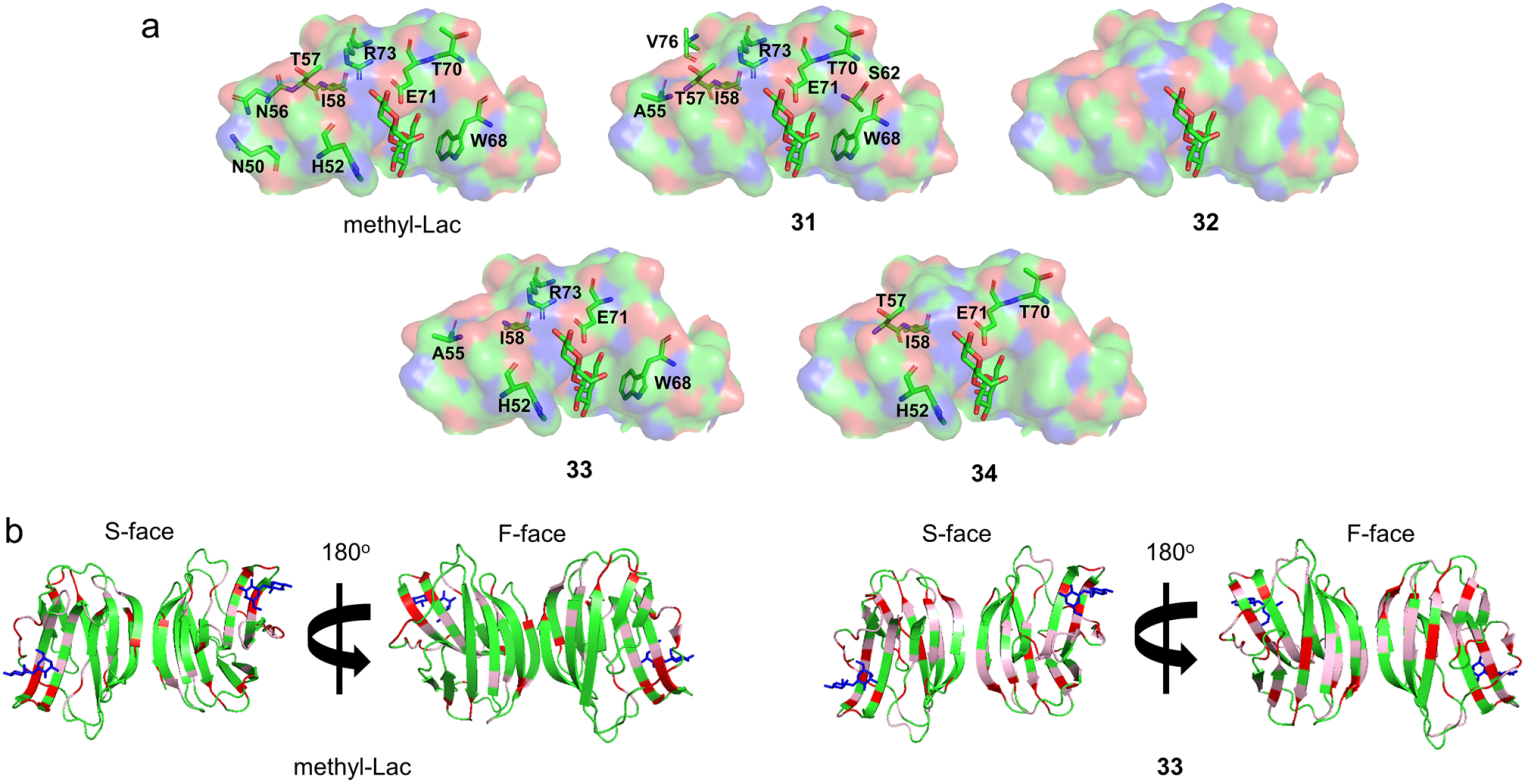
S-face focused electrostatic map of Gal-1 monomer (PDB access code: 1GZW) with the most perturbed amino acid residues by methyl-Lac, glyco-amino acid **31**, peptide **32**, LacNAc-terminated glycopeptide **33,** and sialyl-LacNAc-terminated glycopeptide **34** (**a**). The crystal structure of Gal-1 (PDB access code: 1GZW) is shown with the largest Δδ values highlighted in red (Δδ ≥ 0.05) and pink (0.025 ≥ Δδ < 0.05) for methyl-Lac (**C**) and compound **33** (**b**). For orientation, lactose is presented in all models.

Comparing the perturbation pattern of the S-face of Gal-1 with methyl-Lac and glyco-amino acid **31**, the latter showed a relatively large deviation in the S6 region (W68, T70, E71, R73, V76, and F77) (Figs. 6b,c and 7a; and Supplemental Fig. S13). The perturbation in the S6 region became more pronounced with the addition of LacNAc-terminated glycopeptide **33**, and the perturbation range was found to extend significantly to the F3–F4 regions (V87, C88, F91, D92, and L96) (Fig. 6e and Supplemental Fig. S13). This mechanism is different from the hydrophobic interaction of Gal-1 with protein λ5 of the pre-BCR^54,55^ and CXCL4^56,57^. Interestingly, perturbation with amino acid residues on the L4 region was also significantly enhanced in the presence of glycopeptide **33**. As a result, the aromatic side chains of H52 and W68 recognized galactose, and the ionic couple of E71 and R73 interacted with 3-OH and N-acetyl groups of GlcNAc residue became more prominent (Figs. 6e and 7a; and Supplemental Fig. S13). This indicated that glycopeptide **33** could simultaneously interact with both the S- and F-face of Gal-1 via the S6 intermediate loop (Figs. 6e and 7a,b; and Supplemental Fig. S13). Contrastingly, **34** was predicted to lose its interaction with galactose and W68 because of the presence of sialic acid, as a result of pronounced deviation of the central T57 and adjacent I58 on the S5 region, which impacted the surrounding areas S4–L6 and F3–F4 loop (L8) regions (Figs. 6f and 7a; and Supplemental Fig. S13). The residue I58 is responsible for the inter-protein hydrophobic interaction between S- and F-face showed significant perturbations in all the experiments except for unglycosylated peptide **32**.

Overall, our chemical shift perturbation mapping experiments agreed with our microarray results, where Gal-1 demonstrated high affinity to LacNAc-terminated core M1 glycopeptides via S- (protein-carbohydrate interaction) and F-face (protein-peptide interaction), whose binding was decreased upon extension with sialic acid. Although Gal-1 presented affinity to glycan-free α-DG peptide and LacNAc-terminated core M1 glyco-amino acid in the microarray study at nanomolar range, the NMR experiments suggest that these ligands exhibit binding above the millimolar range. This difference readily reflected the unique binding property of Gal-1 on a bulk surface such as a microarray, where ligands were immobilized at a high density for which *cis*-binding is likely to occur^58^.

### Gal-1 trans-bridges α-DG core M1 glycopeptides and laminins in microarray

Preclinical studies revealed that exogenous application of Gal-1 is an exciting therapeutic potential for treating certain forms of muscular dystrophy^59,60^; however, the precise mechanisms underpinning this phenomenon remain unclear^13^. In the basal lamina, Gal-1 binds to the poly-LacNAc structures of laminins (K_d_≈10^−6^ M), to maintain glycan recognizing function of Gal-1 in the ECM^61^. During muscle repair, Gal-1 binds directly to laminin and α7β1 integrin modulating the late myoblast fusion^20^. In addition, Gal-1 can dissociate from laminin and cross-link olfactory neurons, forming neuronal aggregates in vitro^24^. Laminins is a mutlidomain major components of the basal lamina that anchor cell membrane receptors, such as matriglycan of α-DG, playing pivotal role in establishing cellular stability^7,62^. Notably, hypoglycosylation of the core M3-related structures induces severe disruption of DG-laminin binding in patients with muscle-eye disease (MEB) and Fukuyama congenital muscular disorder (FCMD)^63^. Here, we investigated whether non-labeled Gal-1 could trans-bridge various fluorescently labeled laminins to core M1 α-DG glycopeptides immobilized on microarray slide.

**Figure 8:**
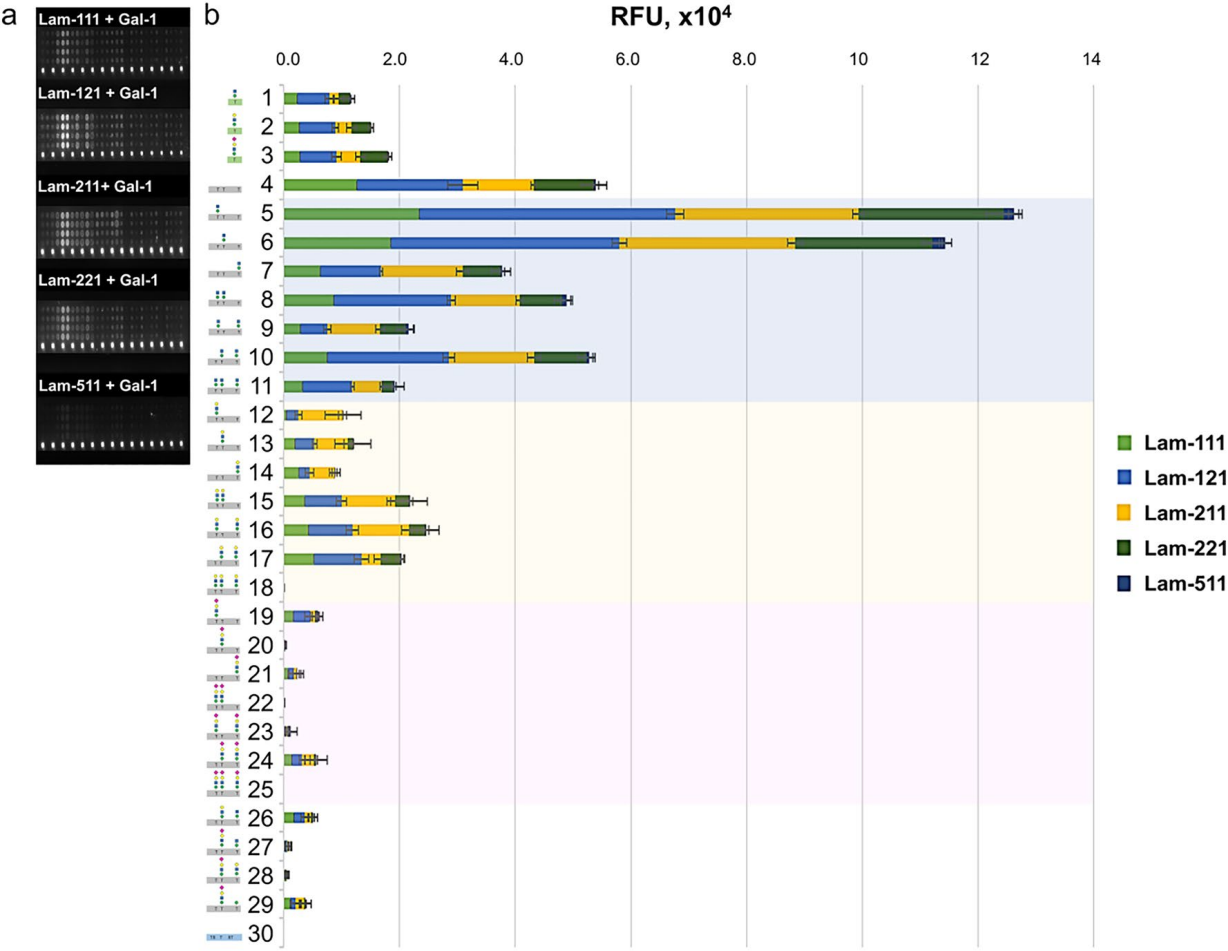
Fluorescence image of microarray chip (**a**) and stacked chart of signal intensities (**b**) of 200 μM core M1 α-DG glycoconjugates taken after treatment with 32.0 μg/mL Cy3-laminin–unlabeled-Gal-1 solution for 30 min. The relative interaction of 200 μM α-DG unglycosylated peptides with 0.10 μg/mL to 32.0 μg/mL Cy3-laminin–unlabeled-Gal-1 solution is in Supplementary Fig. S14.

As previously reported, laminin-111 and -211 could not interact with the α-DG core M1 glycopeptides at pH 7.4^26^. In this study, Gal-1 revealed trans-bridging capabilities, linking laminin-111, -121, -211, and -221 (but little -511) and core M1 α-DG glycopeptides (Fig. 8 and Supplemental Fig. S14). Gal-1 exhibited low laminin-bridging capabilities with glyco-amino acids **1**–**3**. Interestingly, the unglycosylated α-DG peptide **4** and core M1 α-DG glycopeptides (**5**–**11**), could anchor laminins in the presence of Gal-1. The position (**5** vs. **6** vs. **7**) and density (**5**–**7** vs. **8**–**10** vs. **11**) of the core M1 structure along the peptide scaffold affected the trans-bridging activity. This can be attributed to the hydrophobic interaction of Gal-1 with ligands **4**–**11**, which was not dependent on the S-face of the CRD, as discussed previously. In Gal-1 homodimer, the F-face and the canonical S-face sites are oriented on the same side, respectively (Fig. 7b). This structural feature of the Gal-1 homodimer is expected to allow simultaneous interaction with *N*-glycans on the laminin at the S-face and with α-DG peptide at the F-face. The remarkable signal intensities demonstrated by compounds **5** and **6**, which did not show significant interaction with Gal-1, suggest that the *N*-glycan ligand interaction of laminin at the Gal-1 S-face induced a structural and property change in F-face, as observed in NMR study. This result is consistent with the enhanced interaction of Gal-1 with compounds **5** and **6** upon addition of methyl-Lac (Fig. 5b). The previously reported conformational change in the peptide backbone associated with the addition of core M1 could also contribute to this activity change^26^. This unique trans-bridging activity of Gal-1 was also observed during the pre-B/stromal cell synapse formation, where Gal-1 binds to pre-B cell λ5 (via protein–protein interaction) and integrins (via protein-carbohydrate interaction), driving the clustering and activation of pre-B cell receptors^64^.

A decrease in the trans-bridging activity of Gal-1 was observed upon galactosylation of the core M1 structures (**12**–**18**, **28**). In this case, the two S-face of Gal-1 homodimer preferentially bind to poly-LacNAc units of laminins (*cis*-binding) rather than one S-face to LacNAc unit of laminin and one S-face to LacNAc-terminated core M1 α-DG glycopeptide (trans-bridging). Further extension of the LacNAc core M1 structures with sialic acid (**19**–**25**, **27**–**29**) completely abolished the trans-bridging activity of Gal-1 with laminin.

Among the evaluated laminins, laminin-511 was not trans-bridged by Gal-1 to the α-DG peptide and core M1 glycopeptides. The α-chain of laminins possesses a unique structure and is the most glycosylated compared to β- and γ-chains^65,66^. This result indicates that different profile or levels of glycans is present in different α-chain of laminin isoforms which is necessary for the trans-bridging activity of Gal-1 with the prepared ligands. However, the participation of other poly-LacNAc structures located in the β- and γ-chains cannot be neglected.

## Conclusions

Core M1 structures are one of the major expressed glycan of DG whose functional role is less explored. Combining the availability of the array platform with a core M1-type glycopeptide library and a panel of adhesion/growth-regulatory galectins, the binding was detected and the profiles were mapped. The type of glycan, position, and density along the α-DG peptide scaffold, as well as the galectin architecture, determined this binding event. Human Gal-1, -4, and -9 (except -3) can strongly recognize *O*-Man LacNAc-terminated glycoconjugates, making their respective in situ contacts possible via *cis*-binding. The presence of an α2,3-sialylated terminus led to a major reduction in the affinity of galectin, suggesting that this type of extension can fine-tune galectin activity towards this type of *O*-Man glycans. These interactions were significantly inhibited by lactose, establishing that the α-DG core M1-type glycans bind to the canonical sugar-binding site (S-face) of galectin, thus serving as a receptor for galectins.

The interaction with the α-DG peptide and core M1 presenting α-DG glycopeptides, which was not entirely inhibited by lactose, strongly implies that this galectin interaction occurs via hydrophobic interactions that are not dependent on the S-face of galectins. Based on our microarray findings, the ^1^H-^15^N HSQC NMR data showed that LacNAc-terminated core M1 glycopeptide could interact with the S- and F-face of Gal-1, which was diminished by the α2,3-sialylation of this glycoconjugate. These results indicate that galectins can bind with the core M1 glycopeptides of α-DG via peptide- and carbohydrate-protein interactions. Furthermore, we have demonstrated Gal-1 can effectively trans-bridge various laminins (111, 121, 211, and 221, but not 511) to the α-DG peptide and core M1 glycopeptides in the array. This Gal-1 crosslinking activity was completely abrogated by the α2,3-sia extension of the LacNAc core M1 glycopeptides, indicating that Gal-1 binding to the poly-LacNAc side chains of laminin is preferred via *cis*-binding over trans-bridging to α-DG core M1 sialyl-LacNAc ligands.

**Figure 9:**
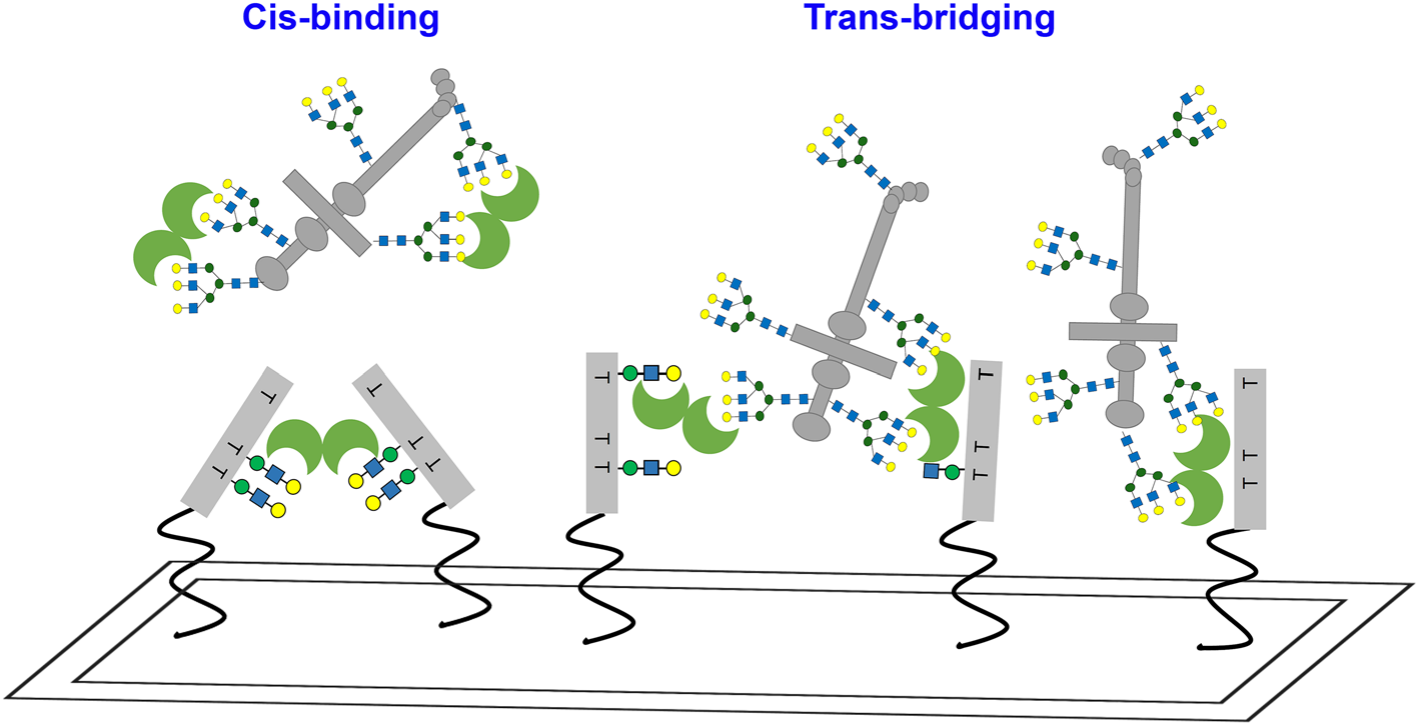
The *cis*-binding and trans*-*bridging activities of Gal-1 with α-DG core M1 glycoconjugates and laminin in situ.

Our experimental setup allowed us to detect the cis/trans-bridging activities of galectins with α-DG glycopeptides and laminin in situ (Fig. 9). However, cellular and in vivo experiments are required to validate these proposed biological mechanisms. Given the co-localization of galectins in the ECM and high interaction of laminin with properly glycosylated α-DG, the investigation of possible binding of galectin-α-DG interaction is an attractive undertaking. Moreover, Gal-1 is highly implicated as a potential therapeutic strategy in treating some form of muscular dystrophy. This inspires further analysis of the (patho)physiological functions of galectin–core M1 α-DG interactions, specifically in α-DGpathy.

## Materials and methods

### Construction of α-dystroglycan mucin type core m1 (glyco)peptide library^26,27^

The α-DG mucin-type core M1 glycoamino acids and glycopeptides were synthesized by a combination of solid-phase synthesis and enzymatic sugar elongations. Each product was purified by a preparative RP-HPLC and was identified by MALDI-TOFMS. Detailed protocol is describe in detail in Supplementary information.

### Preparation and labeling of galectin test panel^67–69^

Galectins were prepared by recombinant expression using the *E.coli* strain BL21 (DE) pLysS and the pGEMEX-1 vector (Promega, Walldorf, Germany). Galectin expressing bacteria were grown at 37 °C until an OD_600_ of 0.6–0.8. Then, the expression of galectin-1 was induced with 100 µM isopropyl ß-D-1-thiogalactopyranoside (IPTG) for at 37 °C, of galectin-3 with 100 µM IPTG at 22 °C, of galectin-4 with 75 µM IPTG at 30 °C and, of galectin-9 with 100 µM IPTG at 22 °C. Induced bacteria were grown for 16 h. Proteins were purified after cell lysis by sonification (three times, each 1 min) through affinity chromatography on a self-made lactose-sepharose resin. Afterwards, bound proteins were eluted from the resin using 20 mM PBS, pH 7.2 containing 50 mM lactose. PBS was replaced by means of a PD10 column with 10 mM sodium carbonate pH 8.5, and the proteins (2–3 mg/mL) were directly conjugated in the dark and in the presence of activity-preserving 20 mM lactose, to NHS-ester Alexa 555 fluorescent dye at 25 °C for 4 h. Unbound dye was removed by gel filtration with a Sephadex G-25 column. Protein purity was checked by gel electrophoresis and Western blotting and maintenance of activity by solid-phase, cell and agglutination assays. Labeled proteins were lyophilized in 50 µg aliquots and stored at − 20 °C until reconstitution. A 1 mg mL^−1^ stock probed galectins were prepared in 1 × PBS (pH 7.40), containing 1% (w/v) BSA, 0.09% (w/v) NaN_3_, and 50% (w/v) glycerol for binding assay and stored at − 20 °C.

### Lectin binding assay^27,70^

Following our reported optimized protocol, the binding profile of lectins were investigated using evanescent-field fluorescence-assisted microarray technology to directly monitor the interaction without the wash and dry-up process and rapid exchange of protein solution to evaluate concentration dependence, 0.10–32.0 µg/mL protein. The AO/PC-copolymer microarray slides were deprotected using 2N HCl treatment overnight at rt, rinsed with MilliQ H_2_O, and dried by centrifugation. Then, test compounds were robotically printed using Arduino-based CNC machine handcrafted robot in quadruplets at two concentrations (20 and 200 µM) in 25 mM AcOH-Pyr (pH 5.0), 0.0025% (w/v) Triton X-100 under constant condition (temperature: 20–25 °C and humidity: 35–45 RH). A 25 µg/mL Cyanin3-keto-BSA (Cy3-keto-BSA) was also printed in the slide as grid. Subsequently, the slide was incubated at 80 °C for 1 h to complete the oxime bond formation. Washed once with Milli-Q H_2_O and dried by centrifugation at 2,000 rpm for 2 min.

A silicon rubber sheet with six chambers was attached to the printed slide. Next, slide was pretreated with reaction buffer {Phosphate-Buffered Saline solution (PBS, 1X) [10 mM Na_2_HPO_4_, 1.8 mM KH_2_PO_4_, 2.7 mM KCl, 137 mM NaCl] pH7.4 containing 0.05% (v/v) Tween-20} for 15 min and dried by centrifugation. The PBS (with or without 10 mM DTT) solutions of galectin were prepared and maintained at a cold ice bath before use. Thereafter, a cover glass was set on each chamber and 12 µL of 0.10 µg/mL lectin solution in reaction buffer was added through the gap of the slide and cover. After 1 h of incubation with the lectin solution at rt in a humidified chamber, the slide was rinsed with washing buffer (reaction buffer with containing 0.05% (v/v) Tween-20) and fluorescence intensity was measured with GlycoStation System. To determine whether additional interactions will be detected at higher lectin concentrations, the reaction buffer solution was carefully removed and replaced with the next lectin test concentration, incubated for 5 min at rt, washed with reaction buffer and then fluorescence intensity was obtained. This step was repeated until all the chosen test concentrations were completed (0.32-, 1.00-, 3.20-, 10.0-, and 32.0 µg/mL).

Images of slides were captured in the presence of reaction buffer. The fluorescexcelence intensities obtained were analyzed using ArrayVision 8.0 (GE Healthcare). For each spot, background correction was applied to get the net intensity, and the average relative fluorescence unit (RFU) was plotted using Microsoft Excel for Microsoft 365. The lectin specificity was identified from high and low RFU values, the error bars being the standard deviation.

### Inhibition experiment

A 12 µL of 10 µg/mL galectin with 2.00 µM of sugar inhibitors (methyl-Lac, cellobiose, maltose) or 100 µM acetylated peptide **4** in PBS was added to the printed test compounds. After 30 min of incubation with the galectin-inhibitor solution at rt in a humidified chamber, the slide was rinsed with washing buffer and fluorescence intensity was measured with GlycoStation System. To determine the effect of higher concentrations of inhibitors, the reaction buffer solution was carefully removed and replaced with the next galectin-inhibitor solution test concentration, incubated for 5 min at rt, washed with reaction buffer and then fluorescence intensity was obtained. This step was repeated until all the chosen inhibitor test concentrations were completed: 20.0 μM, 200 μM, 2.00 mM, 20 mM, and 200 mM for sugar inhibitors; and 200 μM, 500 μM, 1.00 mM, 2.00 mM and 5.00 mM for acetylated peptide **4**. Slide images were captured and analyzed as described above.

### Preparation of ^15^N-enriched Gal-1

^15^N Gal-1 was expressed in *Escherichia coli* cells BL21(DE3) from pET-GST/TEV-LGVLFQGP-hLGALS1 vector (VectorBuilder, Japan). The component cells were transformed with an expression vector utilizing the heat shock method. After one night of incubation on LB Agar plate (containing 100 μg/mL ampicillin), a colony harboring the expression construct was selected and then inoculated unto 5 mL Luria Broth (LB) with antibiotic overnight at 37 °C with shaking. The bacterial pellet was then resuspended to a fresh 500 mL M9-labeled (^15^N-NH_4_Cl as nitrogen source) medium containing ampicillin. After that, culture was grown at 37 °C for at least 4 h until OD_600_ = 0.6–1.2. The cells were then induced with 0.5 mM isopropyl-β-D-1-thiogalactopyranoside (IPTG) overnight at 25 °C. Bacteria expressing ^15^N Gal-1 was lysed with a sonicator (for 5 min, 25% duty cycle. 5 output control) in buffer containing 50 mM PBS, pH 7.4, 150 mM NaCl, 2 mM DTT. 15N Gal-1 was purified using GSH Sepharose beads, cleaved with HRV3CC protease, dialysis, and gel filtration chromatography. Lectin purity as checked by 1D SDS-PAGE. Lectin-binding activity was analyzed by 15N-HQSC with methyl-Lac. The oxidized form of Gal-1 was also prepared using the same protocol without the presence of DTT.

### NMR experiments

The ^15^N-labeled Gal-1 was prepared at 100 µM concentration in 20 mM potassium phosphate buffer at pH 6.9, 50 µM EDTA and 10 mM DTT, made up using H_2_O/D_2_O (95:5%). A 0.05 up to 64 equivalents of ligands were titrated with Gal-1. The ^1^H-^15^N Heteronuclear single quantum coherence (HSQC) NMR experiments were carried out at 25 °C on Bruker 600 AVANCE spectrophotometer equipped with QXI probes and z-axis pulse field gradient units. A gradient sensitivity-enhanced version of the two-dimensional ^1^H-^15^N HSQC experiment (128 scans per transient) was applied with 256 (*t1*) × 2048 (*t*2) complex data points in ^15^N and ^1^H dimensions, respectively. Raw data were converted using TopSpin 4.1.3 and were analyzed using NMRFAM-Sparky^71^. Chemical shift map (Δδ vs. sequence of Gal-1) was plotted using Microsoft Excel. The perturbation pattern of the Gal-1 (PDB ID: 1GZW) was analyzed and visualized by PyMOL 2.5.0 (http://www.pymol.org).

### Laminin–Gal-1 binding assay

A 12 µL of premixed Cy3-labeled human recombinant laminin (BroadPharm and Biolamina, respectively) and unlabeled-Gal-1 with final concentration of 0.10 µg/mL was added to the printed test compounds. After 1 h of incubation with the laminin-galectin solution at rt in a humidified chamber, the slide was rinsed with washing buffer and fluorescence intensity was measured with GlycoStation System. To determine whether additional interactions will be detected at higher laminin-Gal-1 concentrations, the reaction buffer solution was carefully removed and replaced with the next test concentration, incubated for 5 min at rt, washed with reaction buffer and then fluorescence intensity was obtained. This step was repeated until all the chosen test concentrations were completed (0.32-, 1.00-, 3.20-, 10.0-, and 32.0 µg/mL). For weak interaction, incubation with 32.0 µg/mL of laminin-Gal-1 solution was extended for 30 min. Slide images were captured and analyzed as described above.

## Supplementary Information

Supplementary Information.

## Data Availability

The datasets used and/or analysed during the current study is available from the corresponding author on reasonable request.
