# Supplementary material for: Exploring the In situ pairing of human galectins toward synthetic O-mannosylated core M1 glycopeptides of α-dystroglycan
Source: Sci Rep. 2022 Oct 23;12:17800. doi: 10.1038/s41598-022-22758-0 (PMC9588787; doi:10.1038/s41598-022-22758-0)
Supplement: Supplementary file 1 — Supplementary Information. [file 41598_2022_22758_MOESM1_ESM.pdf]

Supplementary Information for

Exploring the *In situ* Pairing of Human Galectins toward Synthetic *O*-Mannosylated Core M1 Glycopeptides of α-Dystroglycan

Lareno L. Villones Jr.[a], Anna-Kristin Ludwig[b], Hiroyuki Kumeta[a], Seiya Kikuchi[a], Rika Ochi[a], Tomoyasu Aizawa[a], Shin-Ichiro Nishimura[a], Hans-Joachim Gabius[b,*,†], Hiroshi Hinou[a]*

[a]Graduate School of Life Science and Faculty of Advanced Life Science, Frontier Research Center for Advanced Material & Life Science, Hokkaido University, N21, W11, Sapporo 001-0021, Japan

[b]Physiological Chemistry, Department of Veterinary Sciences, Faculty of Veterinary Medicine, Ludwig-Maximilians-University Munich, 82152 Planegg-Martinsried, Germany

*Hans-Joachim Gabius, Physiological Chemistry, Department of Veterinary Sciences, Faculty of Veterinary Medicine, Ludwig-Maximilians-University Munich, 82152 Planegg-Martinsried, Germany, gabius@tiph.vetmed.uni-muenchen.de

*Hiroshi Hinou, Graduate School of Life Science and Faculty of Advanced Life Science, Frontier Research Center for Advanced Material & Life Science, Hokkaido University, N21, W11, Sapporo 001-0021, Japan, hinou@sci.hokudai.ac.jp

[†]Died on August 2, 2021

Classification: *To whom correspondence may be addressed.

Keywords: *O*-mannosylation, α-dystroglycan, core M1 glycoconjugates, galectin.

This PDF file includes:

Table S1
Figs. S1 to 14
SI References

Materials and Methods

Materials

Fmoc-amino acids, Fmoc-TentaGel resins functionalized with Rink amide linker and Sieber Amide linker were purchased from Novabiochem. On the other hand, Fmoc(Ac$_3$GlcNAcβ1→2Ac$_3$Manα)Thr was obtained from Medicinal Chemistry Pharmaceuticals (Sapporo, Japan). Bovine β1,4-galactosyltransferase (β1,4-GalT) and α2,3-sialyltransferase from *Pasteurella multocida* (α2,3-SiaT) were purchased from Sigma Aldrich. Activated N-acetylneuraminic acid (CMP-NANA) and uridine-5′-diphosphogalactose, disodium salt (UDP-galactose) were acquired from Yamasa Corporation (Chiba, Japan). Human recombinant laminins (111, 121, 211, 221 and 511) and Cy3 NHS antibody labeling kit were procured from Biolamina and BroadPharm, respectively. Other commercially available solvents and reagents were purchased from Sigma-Aldrich (USA), Tokyo Chemical Industry (Tokyo, Japan), Wako Pure Chemical Industries (Osaka, Japan), Kokusan Chemical (Tokyo, Japan), or Watanabe chemical (Osaka, Japan) and used without purification, unless stated.

Manual microwave-assisted solid-phase synthesis was carried out in a polypropylene tube equipped with a filter (LibraTube, Hipep Laboratories, Kyoto, Japan). Green Motif 1 microwave synthesis reactor (IDX Corp, Japan) was used for the glycopeptide synthesis with microwave irradiation. The reaction vessel was placed inside the cavity of the instrument and was irradiated with 2,450 MHz single-mode microwave at 50°C, stirred continuously with a vortex mixer. High-performance liquid chromatography system (HPLC; HITACHI, Japan) was used to purify the compounds using a preparative C-18 reversed-phase column (Intersil ODS-3 10×250 mm) equipped with L7150 pump, at flow rate 5 mL/min monitored by UV detector at 220 nm at room temperature. Bruker Daltonics (Germany) Ultraflex MALDI-TOF/TOF mass spectrometer was used for MALDI experiments using DHB as a matrix.

Microarray fluorescence images were obtained using GlycoStation Reader 1200 (GlycoTechnica Ltd., Yokohama, Japan), analyzed by ArrayVision software V8.0 (GE Healthcare, Tokyo, Japan). Background correction was applied to get the net intensity. The average relative fluorescence unit (RFU) was plotted as a histogram, error bars being the standard deviation utilizing Microsoft Excel.

Construction of α-dystroglycan mucin type core m1 (glyco)peptide library[1,2]

The *O*-Man core m1 α-DG peptide (**4**), MUC1 peptide (**30**), glycol-amino acids (**1**) and glycopeptides (**5-11**) were synthesized manually by microwave-assisted solid-phase synthesis by using H-Rink Amide ChemMatrix® (0.48 mmol/g, 24 µmol) resin. The resin was swollen with CH_2Cl_2 for 1 h at room temperature. The protected Fmoc-amino acid (4.0 equiv) was pre-activated by treating with HBTU (4.0 equiv), HOBt (4.0 equiv) and DIEA (6.0 equiv) in DMF (455 µL) for 9 min under microwave irradiation and then attached to the resin. In every step, the N-fluorene-9-ylmethoxycarbonyl (Fmoc) groups at N-terminal were removed by 20% piperidine in DMF (1 mL) for 3 min under microwave irradiation. All coupling reactions were done for 10 min, and solvents were removed using PP syringes fitted with a porous disk. For glycosylated amino acid, Fmoc-Thr(Ac$_3$GlcNAcβ1→2Manα1)-OH (1.2 equiv) was treated with PyBOP (1.2 equiv), HOAt (1.2 equiv) and DIEA (3.0 equiv) in DMF (275 µL) subjected to MW irradiation for 9 min at 50°C. After which, PyBOP-HOAt (1.2 equiv) was added and allowed to react for another 9 min. As the final synthesis step, 5-oxohexanoic acid (3 equiv) was introduced at the N-terminus of each glycopeptidyl resin, according to the above coupling procedure for Fmoc-amino acids. Simultaneous removal of side-chain protecting groups and cleavage of glycopeptide from the resin was performed by treating 95% aqueous TFA (1 mL) for 1 h at ambient temperature. In a cold water bath, the crude peptides and glycopeptides were precipitated by tert-butyl methyl ether (5 mL). After that, the solution was centrifuged at 3000 rpm for 1 min, and the supernatant was carefully removed. The precipitate was dissolved in milli-Q water (5 mL), and lyophilized. Then, deacetylation of the glycan moiety was done by dissolving the lyophilized material in methanol, pH was adjusted at 12.5 with dropwise addition of 1M NaOH, and the solution was stirred at room temperature for 1 h. After the deprotection, the solution was neutralized with 20% AcOH in methanol and a flow of nitrogen gas removed the solvent. The crude peptides and glycopeptides was purified by a preparative RP-HPLC, using a preparative C18- reversed phase column on HITACHI liquid chromatography system (HPLC) at a flow rate of 5 mL per min. Eluent A was distilled water containing 0.1% TFA, and eluent B was acetonitrile containing 0.1% TFA. Each product was analyzed by MALDI-TOFMS.

Compounds **2, 12**–**18** were obtained by galactosylation of compounds **1, 5**–**11** using galactosyltransferase from bovine milk and UDP-Gal in 50 mM HEPES buffer (pH 7.0), 10 mM $MnCl_2$, and 0.1 wt% BSA incubated for 24 h. Subsequently, compounds **2, 12**–**18** were sialylated using α2,3-sialyltransferase from *Pasteurella multocida* and CMP-NANA in 50 mM Tris buffer (pH 6.5) and 500 mM

NaCl incubated for 36 h to yield compounds **3, 19**–**25**. Compounds **26**–**29** were synthesized by solid-phase synthesis and enzymatic sugar elongations, as described earlier. Each product was purified by a preparative RP-HPLC, using a preparative C18- reversed phase column on HITACHI liquid chromatography system (HPLC) with an appropriate solvent system at a flow rate of 5 mL per min and was identified by MALDI-TOFMS.

Table S1. *O*-Man core m1 based glycopeptide sequence utilized in this study (glycosylated positions in **red** and **blue**).

Code	Glycan	Sequence
1	GlcNAcβ(1→2)Manα1→	5-oxo-hexanoyl-PEG-**T**-NH_2
2	Galβ(1→4)GlcNAcβ(1→2) Manα1→	5-oxo-hexanoyl-PEG-**T**-NH_2
3	Siaα(2→3)Galβ(1→4) GlcNAcβ(1→2) Manα1→	5-oxo-hexanoyl-PEG-**T**-NH_2
4	None	5-oxo-hexanoyl-TRGAIIQTPTLGPIQPTRV -NH_2
5	GlcNAcβ(1→2)Manα1→	5-oxo-hexanoyl-TRGAIIQ**T**PTLGPIQPTRV -NH_2
6	GlcNAcβ(1→2)Manα1→	5-oxo-hexanoyl-TRGAIIQTP**T**LGPIQPTRV -NH_2
7	GlcNAcβ(1→2)Manα1→	5-oxo-hexanoyl-TRGAIIQTPTLGPIQP**T**RV -NH_2
8	GlcNAcβ(1→2)Manα1→	5-oxo-hexanoyl-TRGAIIQ**T**P**T**LGPIQPTRV -NH_2
9	GlcNAcβ(1→2)Manα1→	5-oxo-hexanoyl-TRGAIIQ**T**PTLGPIQP**T**RV -NH_2
10	GlcNAcβ(1→2)Manα1→	5-oxo-hexanoyl-TRGAIIQTP**T**LGPIQP**T**RV -NH_2
11	GlcNAcβ(1→2)Manα1→	5-oxo-hexanoyl-TRGAIIQ**T**P**T**LGPIQP**T**RV -NH_2
12	Galβ(1→4)GlcNAcβ(1→2)Manα1→	5-oxo-hexanoyl-TRGAIIQ**T**PTLGPIQPT RV-NH_2
13	Galβ(1→4)GlcNAcβ(1→2)Manα1→	5-oxo-hexanoyl-TRGAIIQTP**T**LGPIQPT RV -NH_2
14	Galβ(1→4)GlcNAcβ(1→2)Manα1→	5-oxo-hexanoyl-TRGAIIQTPTLGPIQP**T**RV -NH_2
15	Galβ(1→4)GlcNAcβ(1→2)Manα1→	5-oxo-hexanoyl-TRGAIIQ**T**P**T**LGPIQPTRV -NH_2
16	Galβ(1→4)GlcNAcβ(1→2)Manα1→	5-oxo-hexanoyl-TRGAIIQ**T**PT LGPIQP**T**RV -NH_2
17	Galβ(1→4)GlcNAcβ(1→2)Manα1→	5-oxo-hexanoyl-TRGAIIQTP**T**LGPIQP**T**RV -NH_2
18	Galβ(1→4)GlcNAcβ(1→2)Manα1→	5-oxo-hexanoyl-TRGAIIQ**T**P**T**LGPIQP**T**RV -NH_2
19	Siaα(2→3)Galβ(1→4)GlcNAcβ(1→2)Manα1→	5-oxo-hexanoyl-TRGAIIQ**T**PTLGPIQPTRV -NH_2
20	Siaα(2→3)Galβ(1→4)GlcNAcβ(1→2)Manα1→	5-oxo-hexanoyl-TRGAIIQTP**T**LGPIQPTRV -NH_2
21	Siaα(2→3)Galβ(1→4)GlcNAcβ(1→2)Manα1→	5-oxo-hexanoyl-TRGAIIQTPTLGPIQP**T**RV -NH_2
22	Siaα(2→3)Galβ(1→4)GlcNAcβ(1→2)Manα1→	5-oxo-hexanoyl-TRGAIIQ**T**P**T**LGPIQPTRV -NH_2
23	Siaα(2→3)Galβ(1→4)GlcNAcβ(1→2)Manα1→	5-oxo-hexanoyl-TRGAIIQ**T**PTLGPIQP**T**RV -NH_2
24	Siaα(2→3)Galβ(1→4)GlcNAcβ(1→2)Manα1→	5-oxo-hexanoyl-TRGAIIQTP**T**LGPIQP**T**RV -NH_2
25	Siaα(2→3)Galβ(1→4)GlcNAcβ(1→2)Manα1→	5-oxo-hexanoyl-TRGAIIQ**T**P**T**LGPIQP**T**RV -NH_2
26	Galβ(1→4)GlcNAcβ(1→2) Manα1→<br><br>GlcNAcβ(1→2)Manα1→	5-oxo-hexanoyl-TRGAIIQTP**T**LGPIQP**T**RV -NH_2
27	Siaα(2→3)Galβ(1→4)GlcNAcβ(1→2)Manα1→<br><br>GlcNAcβ(1→2)Manα1→	5-oxo-hexanoyl-TRGAIIQTP**T**LGPIQP**T**RV -NH_2
28	Siaα(2→3)Galβ(1→4)GlcNAcβ(1→2)Manα1→<br><br>Galβ(1→4)GlcNAcβ(1→2) Manα1→	5-oxo-hexanoyl-TRGAIIQTP**T**LGPIQP**T**RV -NH_2
29	Siaα(2→3)Galβ(1→4)GlcNAcβ(1→2)Manα1→<br><br>Manα1→	5-oxo-hexanoyl-TRGAIIQTP**T**LGPIQP**T**RV -NH_2
30	None	5-oxo-hexanoyl-PEG-GVTSAPDTRPAPGSTAPPAHGVT-NH_2

The synthesis and characterization of compounds used in this study is reported elsewhere[1].

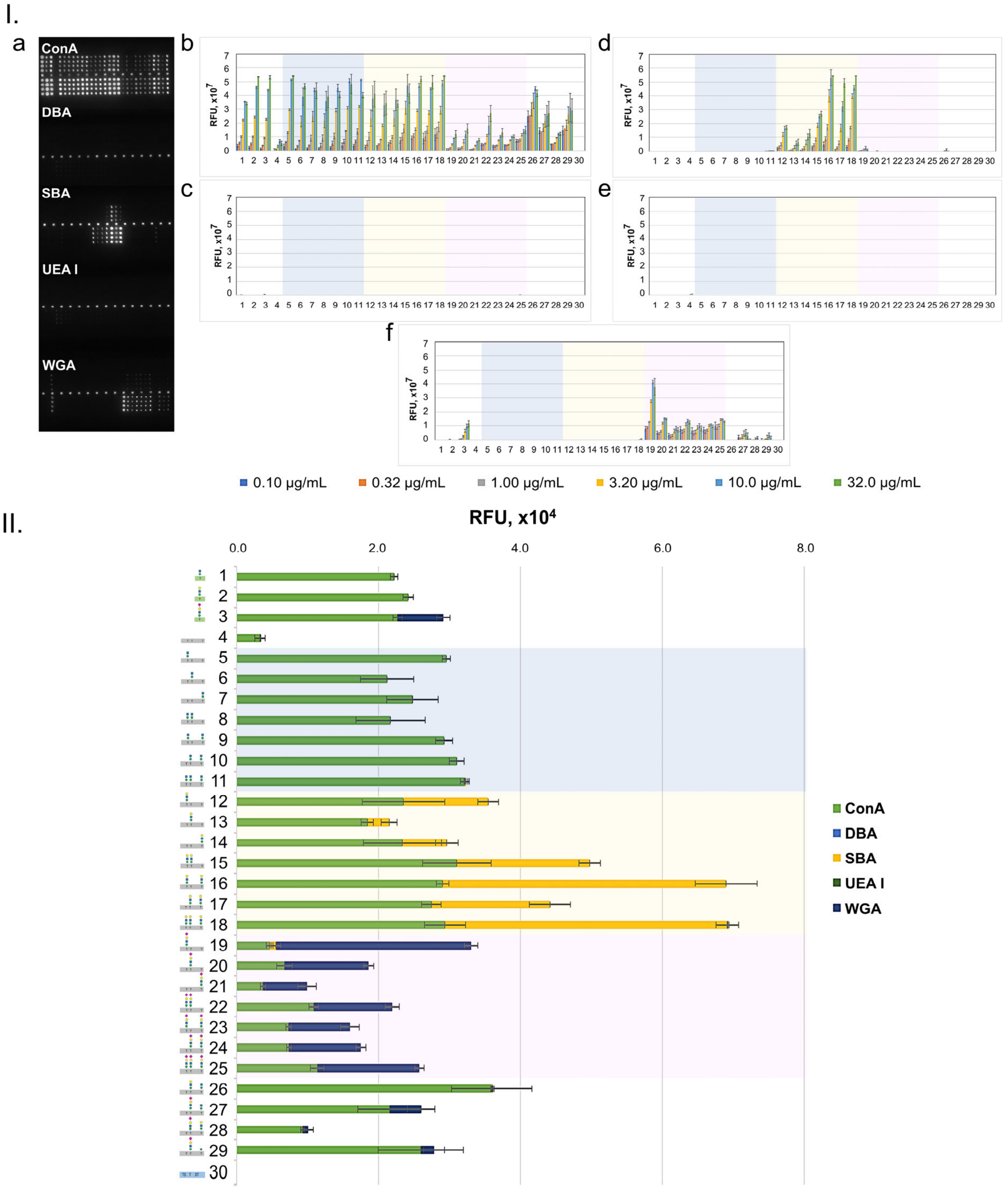

Figure S1. (**I.**) Fluorescence image of microarray chip is taken after treatment of 20 and 200 µM core M1 α-DG glycopeptides with 10.0 µg/mL plant lectin solution (**a**) and relative binding properties of 200 µM core M1 of αDG with 0.10 to 32.0 µg/mL rhodamine-label plant lectins; *Concanavalia enisformis* agglutinin (ConA) (**b**), *Dolichos biflorus* agglutinin (DBA) (**c**), *Glycine max* (Soybean) agglutinin (SBA) (**d**), *Ulex europaeus* agglutinin I (UEA I) (**e**), *Triticum vulgaris* (Wheat germ) agglutinin (WGA) (**f**). (**II.**)Stacked chart of signal intensities of 200 µM core M1 α-DG glycopeptides with 3.20 µg/mL of plant lectins *Concanavalia ensiformis* agglutinin (ConA), *Dolichos biflorus* agglutinin (DBA) , *Glycine max* (Soybean) agglutinin (SBA), *Ulex europaeus* agglutinin I (UEA I), and *Triticum vulgaris* (Wheat germ) agglutinin (WGA).

a

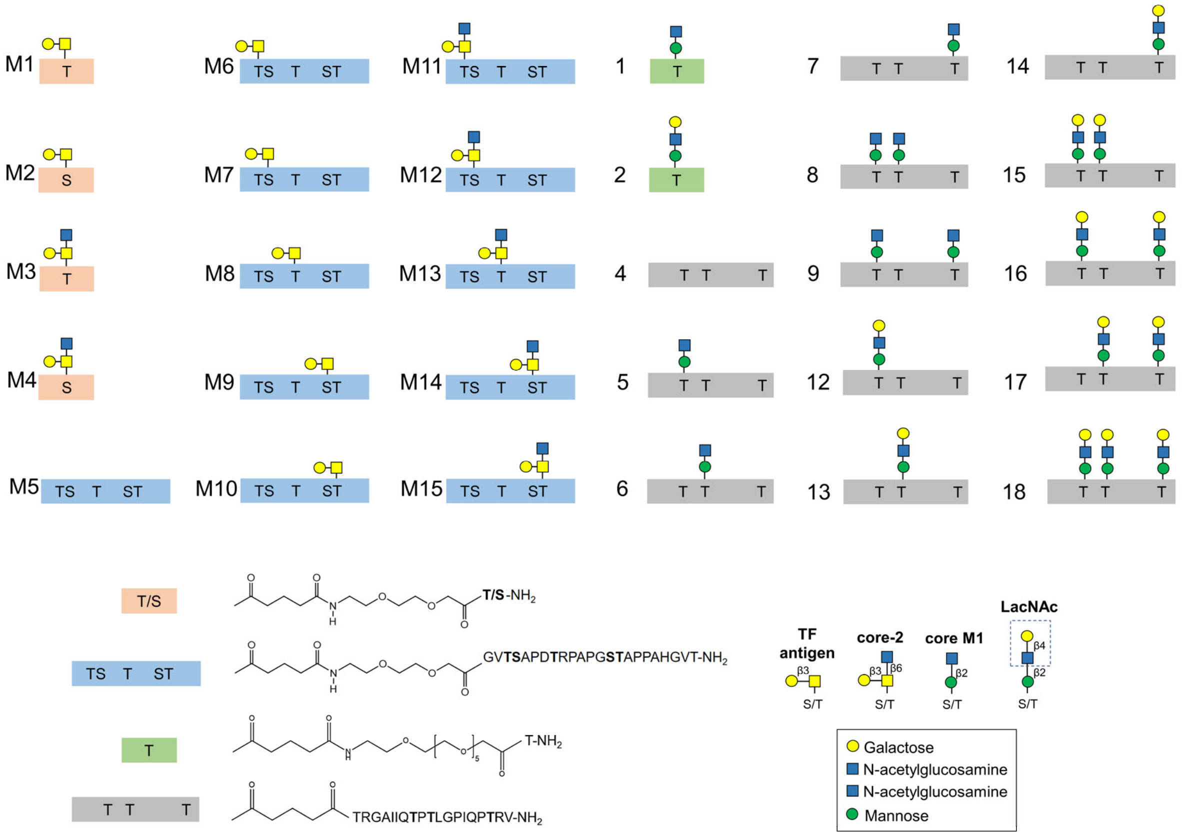

b

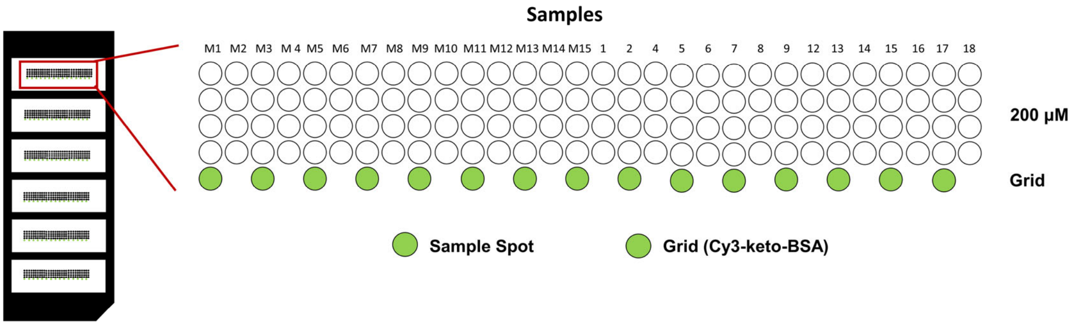

Figure S2. Synthetic MUC1 and α-DG core M1 glycopeptides used for preliminary microarray experiments (**a**). Compounds were robotically printed in quadruplets at 200 µM on an aminooxy- coated plastic slide. Subsequently, a six-chamber rubber silicon sheet was attached. Green spots correspond to cyanine 3-keto-BSA (Cy3-keto-BSA) as grid (**b**).

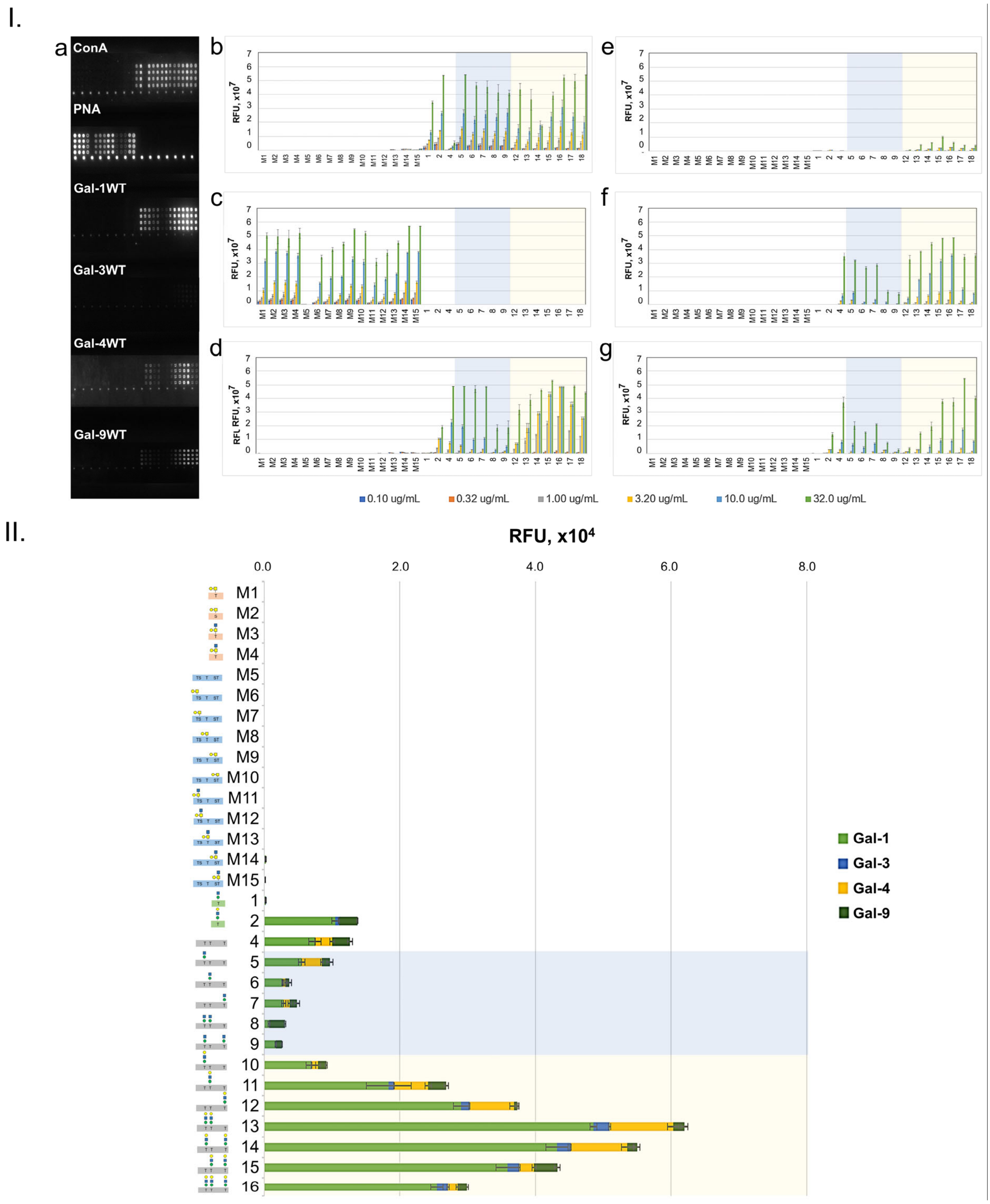

Figure S3. (**I**.) Synthetic MUC1 and α-DG core M1 glycopeptides used for preliminary microarray experiments. (**II**.) Fluorescence image of microarray chip of selected galactose-terminated MUC1 and α-DG glycopeptide peptide library taken after treatment with 10.0 μg/mL galectin solution (**a**) and relative interaction of 200 μM glycopeptides with 0.10 μg/mL to 32.0 μg/mL ConA (**b**), PNA (**c**), Gal-1 (**d**), Gal-3 (**e**), Gal-4 (**f**), and Gal-9 (**g**). (**III**.) Stacked chart of signal intensities of 200 μM of selected MUC1 and core M1 α-DG glycopeptides with 3.20 μg/mL of galectins. The synthesis and characterization of MUC1 glycopeptides used in this study is reported elsewhere[2,3].

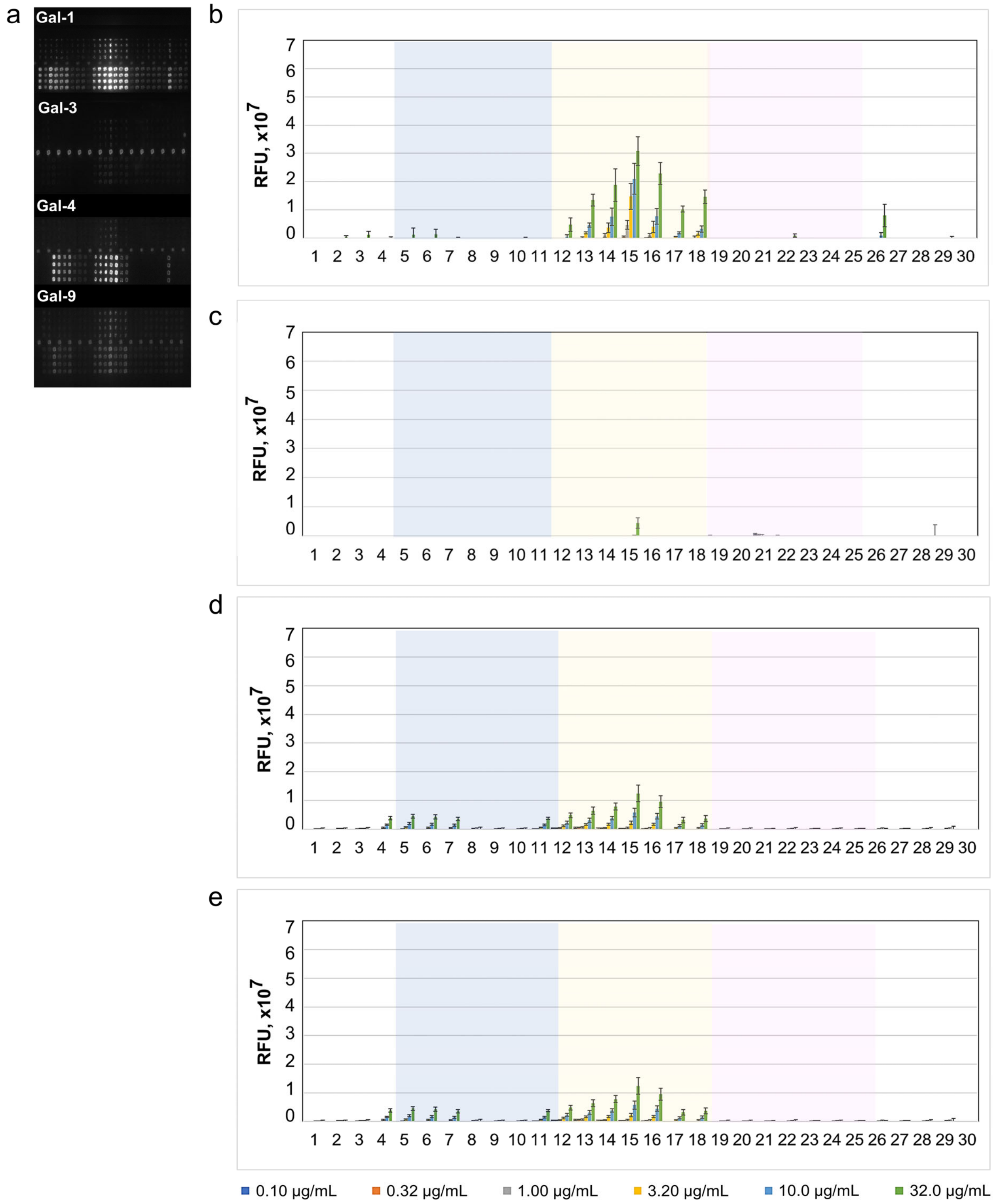

Figure S4. Fluorescence image of microarray chip is taken after treatment of 20 and 200 μM core M1 α-DG glycoconjugates with 10.0 μg/mL galectin solution (**a**) and relative interaction of **20 μM** core m1 α-DG glycoconjugates with 0.10 μg/mL to 32.0 μg/mL Gal-1 (**b**), Gal-3 (**c**), Gal-4 (**d**), and Gal-9 (**e**).

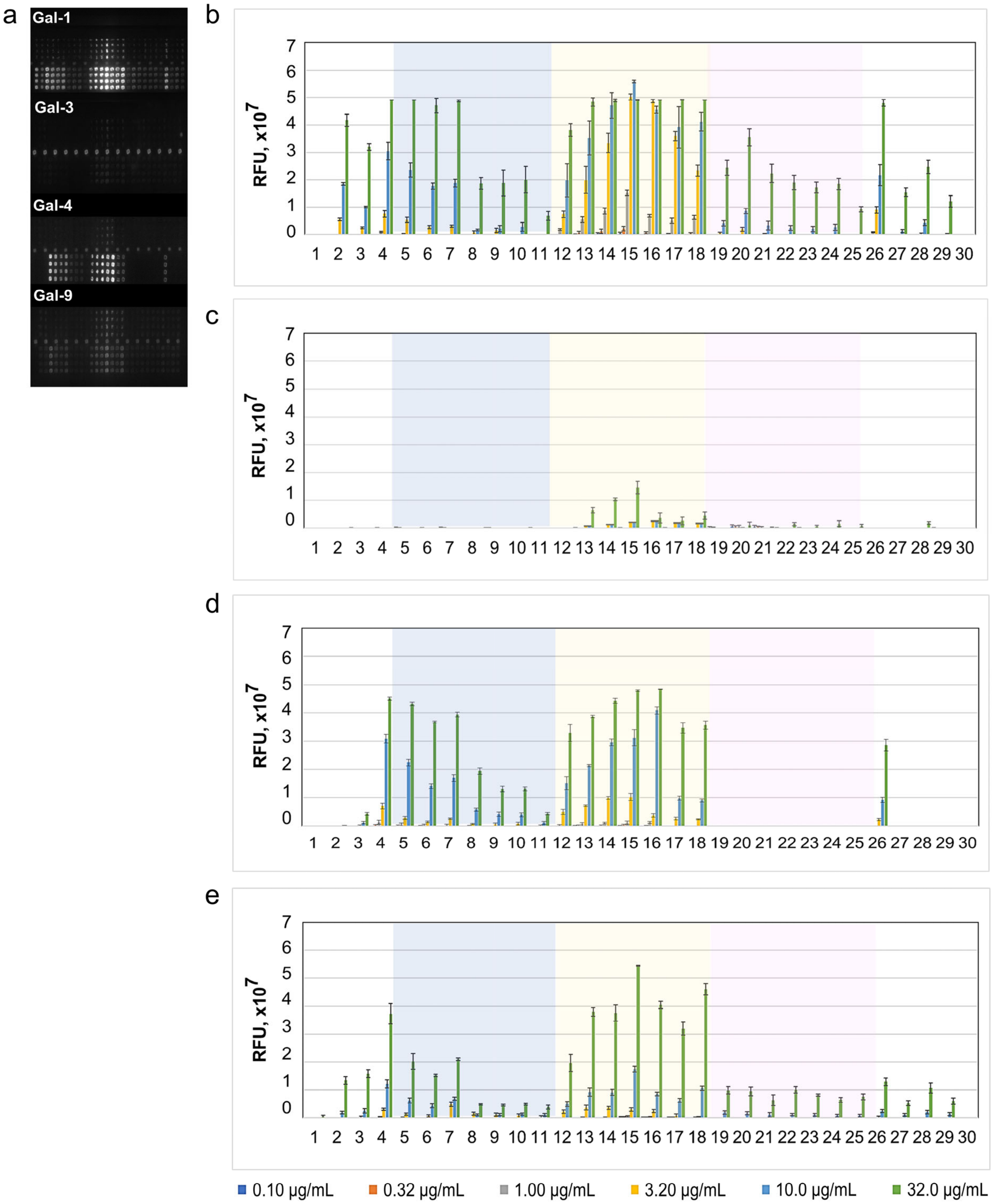

Figure S5. Fluorescence image of microarray chip is taken after treatment of 20 and 200 µM core M1 α-DG glycoconjugates with 10.0 µg/mL galectin solution (**a**) and relative interaction of **200 µM** core m1 α-DG glycoconjugates with 0.10 µg/mL to 32.0 µg/mL Gal-1 (**b**), Gal-3 (**c**), Gal-4 (**d**), and Gal-9 (**e**).

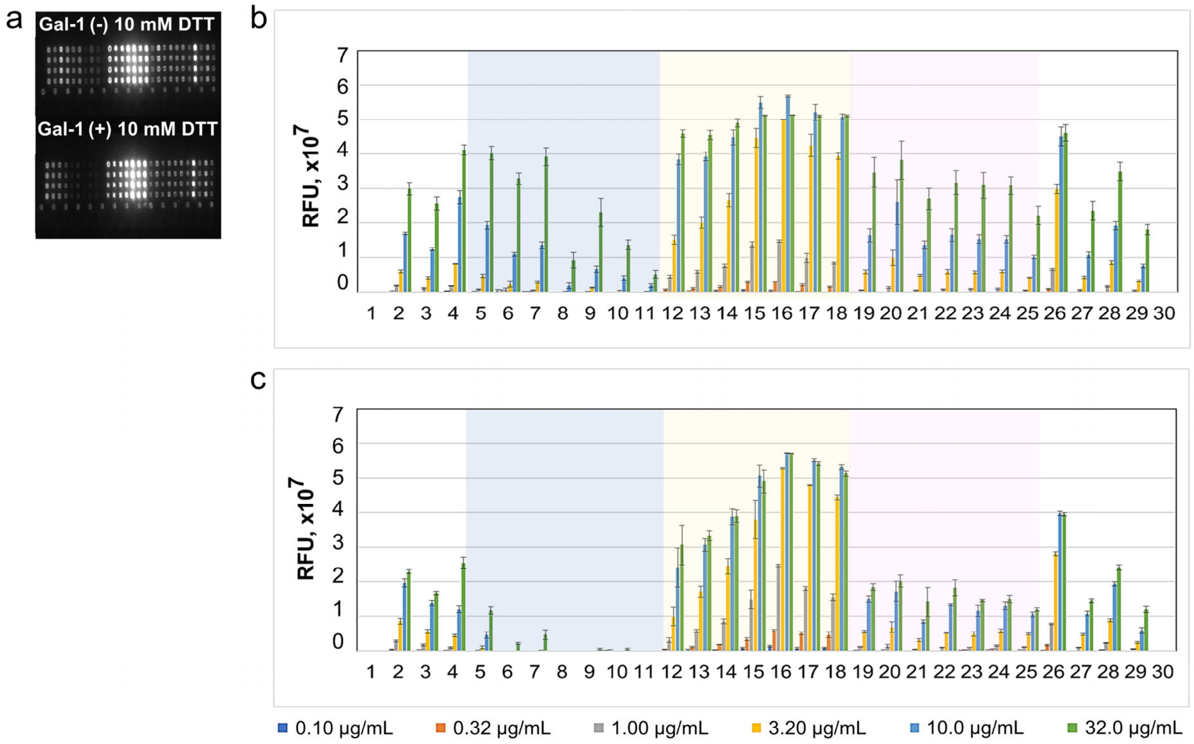

Figure S6. Fluorescence image of microarray chip is taken after treatment of 200 µM core M1 α-DG glycopeptides with 10.0 µg/mL Gal-1 in the absence and presence of 10 mM DTT (**a**) and relative interaction of 200 µM core M1 α-DG glycopeptides with 0.10 µg/mL to 32.0 µg/mL Gal-1 absence (**b**) and presence (**c**) of 10 mM DTT.

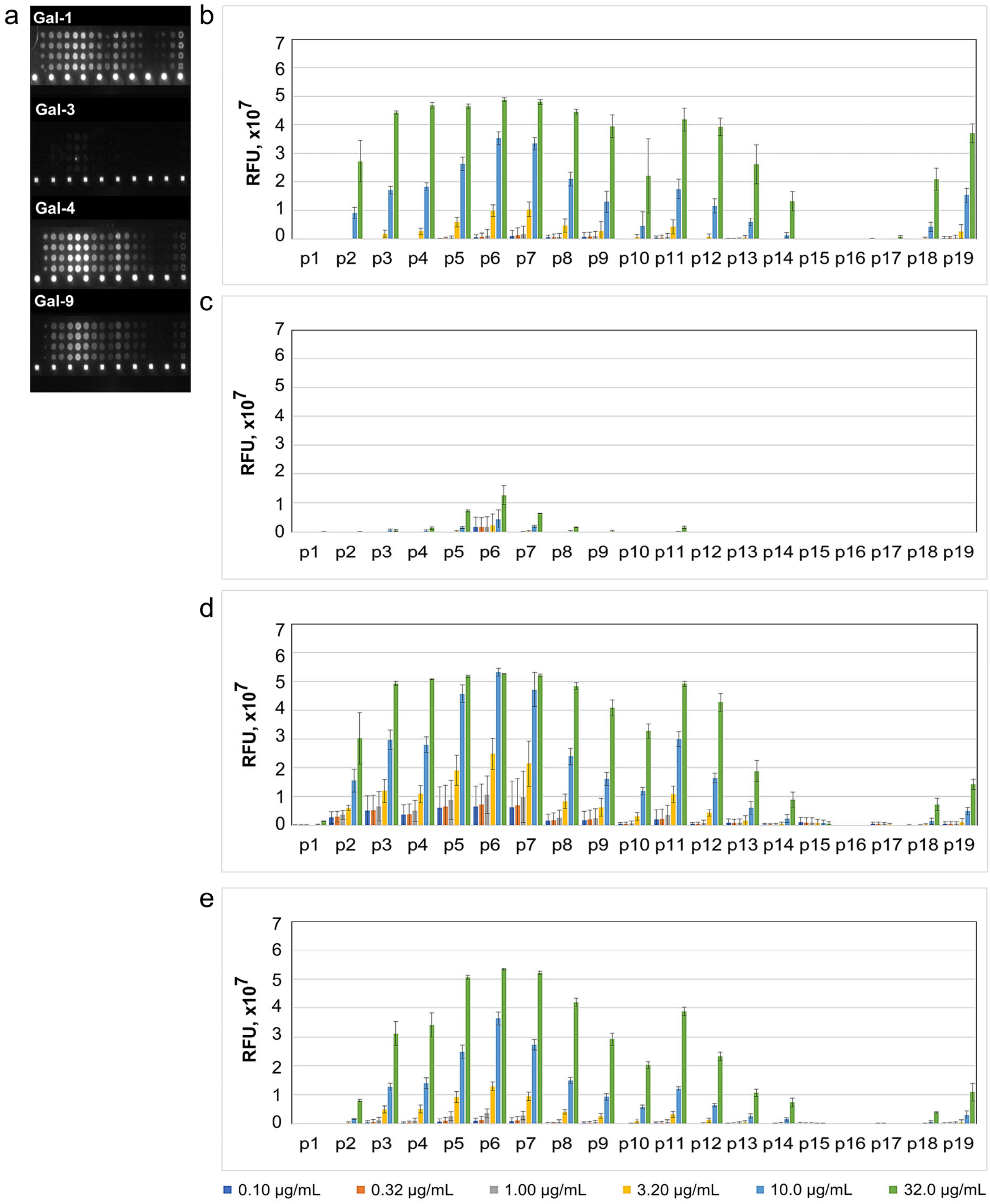

Figure S7. Fluorescence image of microarray chip of α-DG unglycosylated peptide library taken after treatment with 10.0 µg/mL galectin solution (**a**) and relative interaction of 200 µM α-DG unglycosylated peptides with 0.10 µg/mL to 32.0 µg/mL Gal-1 (**b**), Gal-3 (**c**), Gal-4 (**d**), and Gal-9 (**e**).

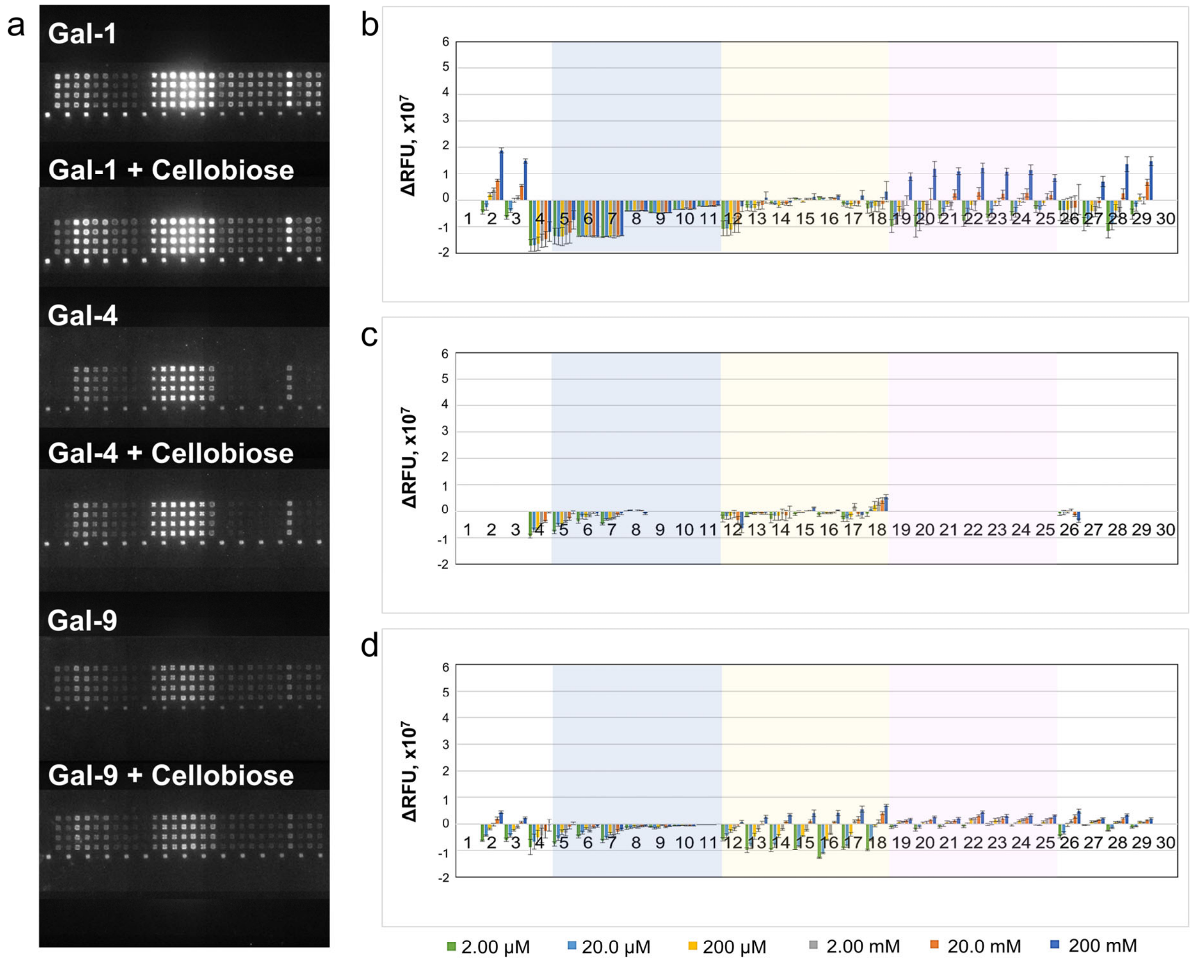

Figure S8. Fluorescence image of microarray chip is taken after treatment of 10 μg/mL lectin solution with 200 mM cellobiose (**a**) and relative binding properties of 200 μM core M1 α-DG glycopeptides with Gal-1 (**b**), Gal-4 (**c**), and Gal-9 (**d**) with the presence of 2.00 μM to 200 mM cellobiose.

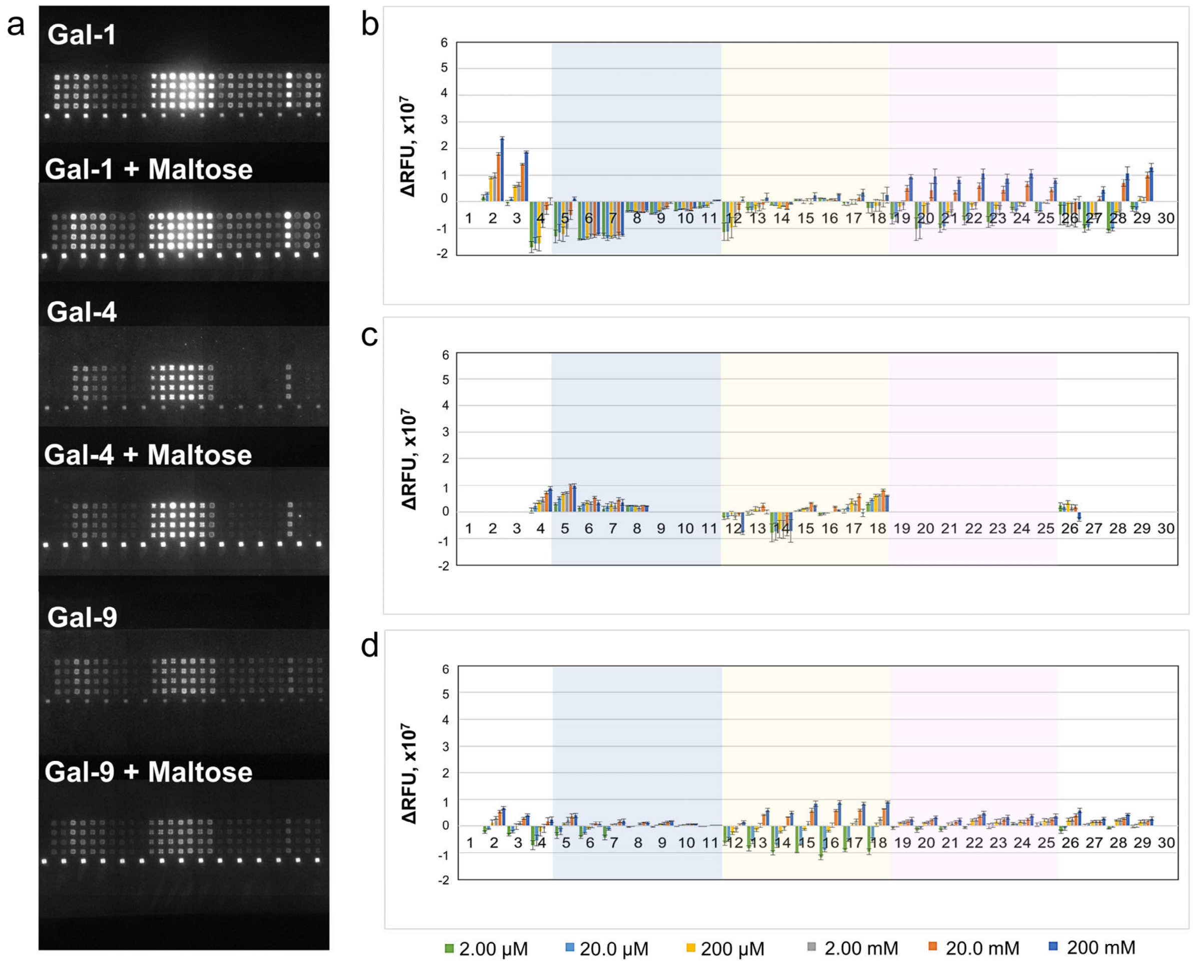

Figure S9. Fluorescence image of microarray chip is taken after treatment of 10.0 μg/mL lectin solution with 200 mM maltose (**a**) and relative binding properties of 200 μM core M1 α-DG glycopeptides Gal-1 (**b**), Gal-4 (**c**), and Gal-9 (**d**) with the presence of 2.00 μM to 200 mM maltose.

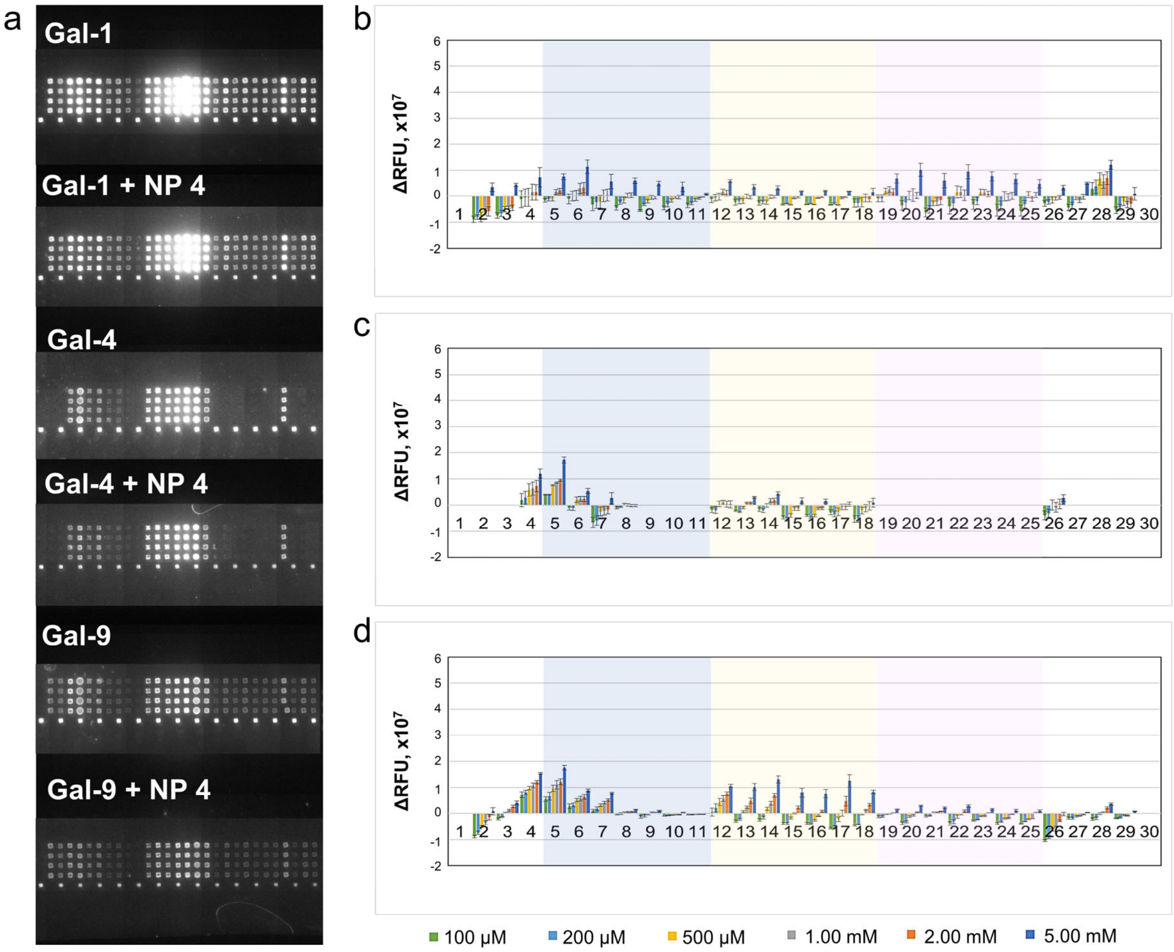

Figure S10. Fluorescence image of microarray chip is taken after treatment of 10 μg/mL lectin solution with 5.00 mM acetylated peptide **4** (**a**) and relative binding properties of 200 μM core M1 α-DG glycopeptides with Gal-1 (**b**), Gal-4 (**c**), and Gal-9 (**d**) with the presence of 100 μM to 5.00 mM acetylated peptide **4**.

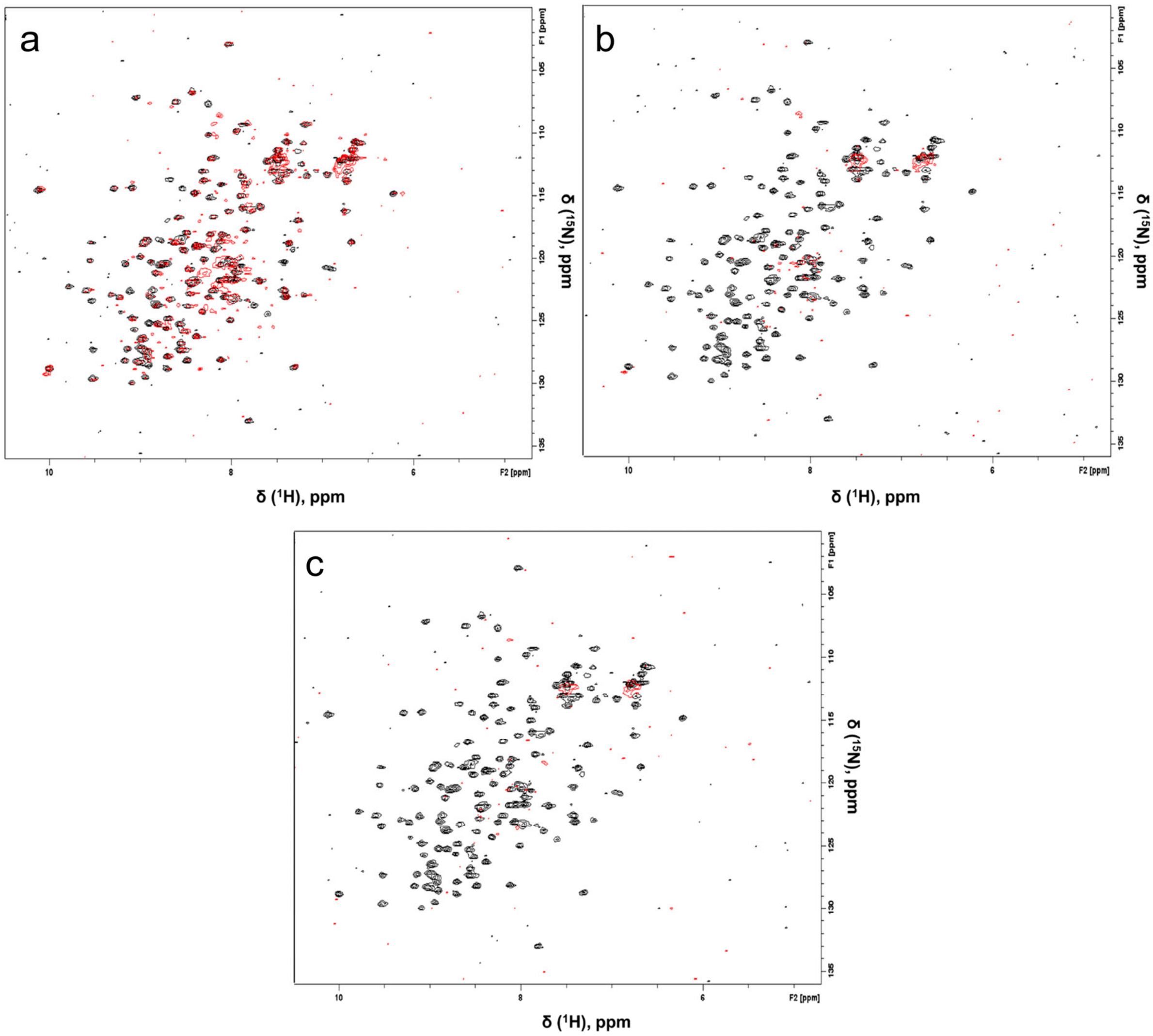

Figure S11. Overlaid ^{1}H-^{15}N HSQC spectra of 100 μM Gal-1$_{reduced}$ (black) with 100 μM Gal-1$_{oxidized}$ (red) using air (**a**), 6.9 μM $CuSO_4$ (**b**), and 1.0 mM tetramethylazodicarboxamide (**c**).

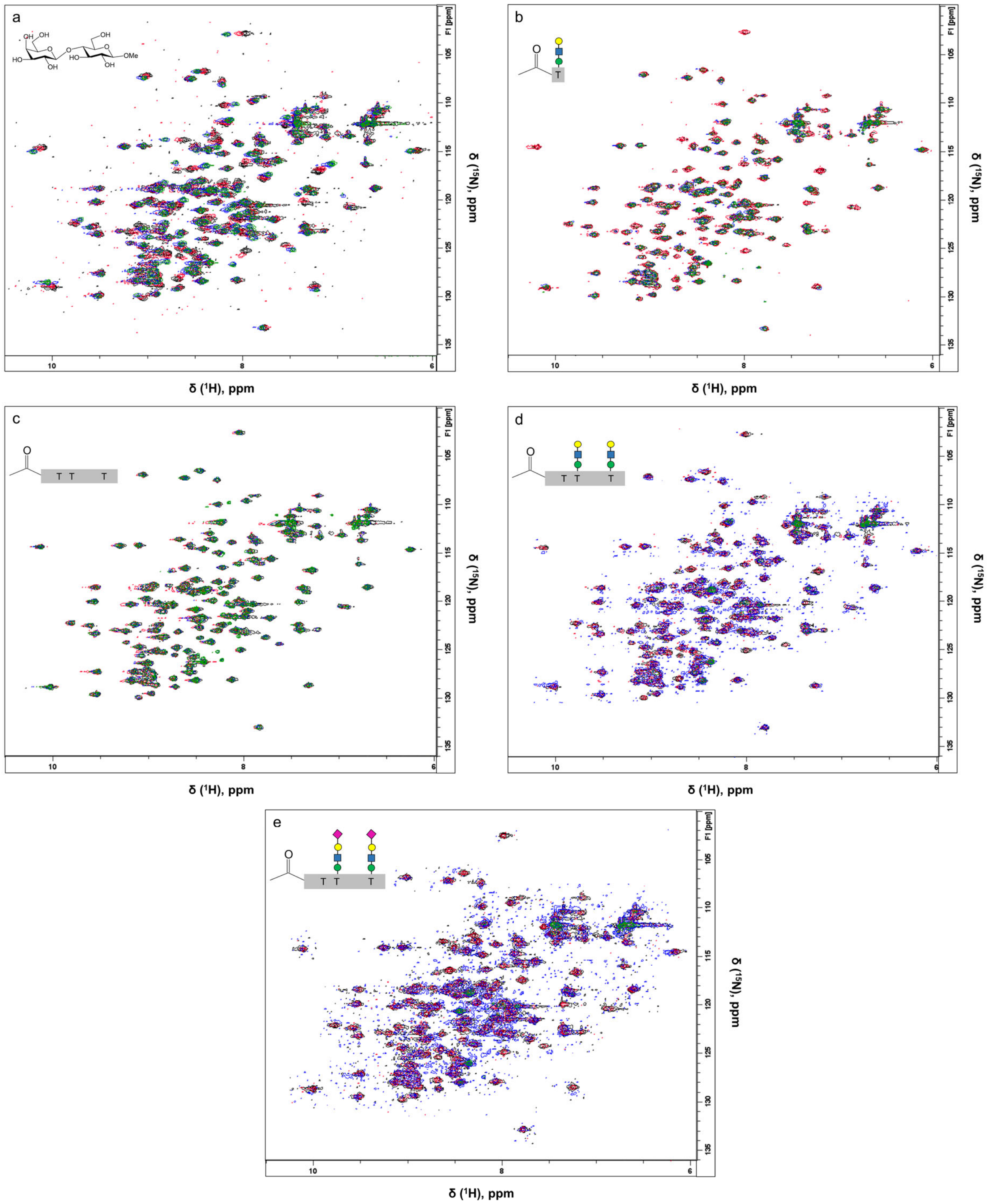

Figure S12. Overlaid ^{1}H-^{15}N HSQC spectra of reduced Gal-1 (100 μM) alone (**black** peaks) and in the presence of methyl-β-lactoside (molar ratio 1:1 **blue** peaks, 1:16 **red** peaks, and 1:64 **green** peaks, **a**), LacNAc-terminated glycoamino acid **31** (molar ratio 1:1 **blue** peaks, 1:32 **red** peaks, and 1:64 **green** peaks **b**), unglycosylated peptide **32** (molar ratio 1:1 **blue** peaks, 1:16 **red** peaks, and 1:64 **green** peaks **c**), LacNAc-terminated glycopeptide acid **33** (molar ratio 1:0.125 **blue** peaks, 1:1 **red** peaks, and 1:2 **green** peaks **d**), sialyl-LacNAc-terminated glycopeptide **34** (molar ratio 1:0.125 **blue** peaks, 1:1 **red** peaks, and 1:2 **green** peaks **e**), Solution condition were 20 mM potassium phosphate buffer, pH 6.9, 50 μM EDTA, with 10 mM DTT made up using a H_2O/D_2O (95:5%).

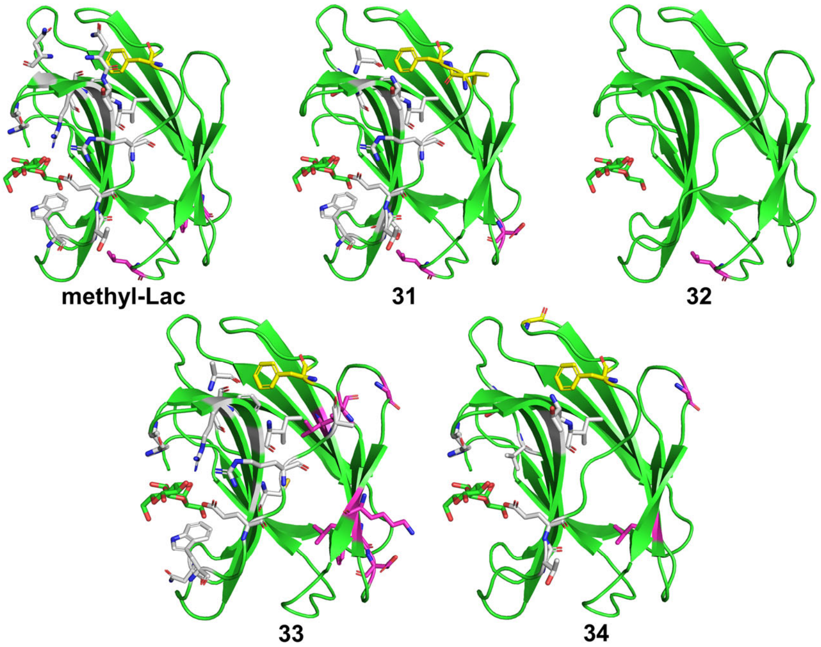

Figure S13. Close-up view of Gal-1 monomer S6 region binding site of methyl-β-lactose, glycoamino acid **31**, nonglycosylated peptide **32**, glycopeptides **33** and **34**. Peptide backbone (green); most perturb amino acid residues in S-Face (gray); most perturb amino acid in F-face (violet). For orientation, lactose (C and O atoms are highlighted in green and red, respectively) is presented.

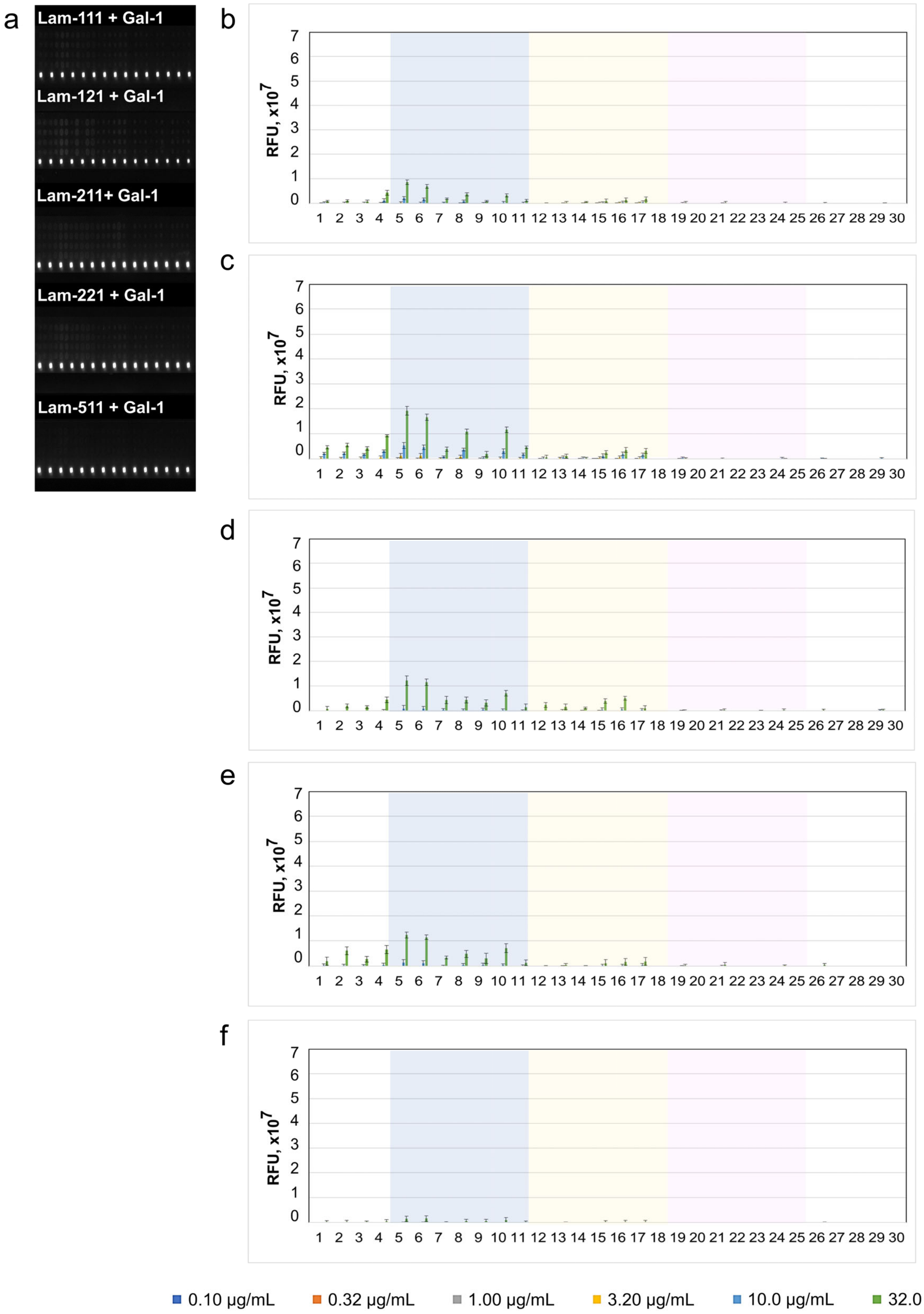

Figure S14. Fluorescence image of microarray chip is taken after treatment of 200 µM core M1 α-DG glycopeptides with 10.0 µg/mL laminin–Gal-1 solution (**a**) and relative binding properties of 200 µM core M1 of αDG with 0.10 to 32.0 µg/mL Laminin-111 and Gal-1 (**b**), Laminin-121 and Gal-1 (**c**), Laminin-211 and Gal-1 (**d**), Laminin-221 and Gal-1 (**e**), Laminin-511 and Gal-1 (**f**).

References

1. Hinou, H. *et al.* Synthetic glycopeptides reveal specific binding pattern and conformational change at O-mannosylated position of α-dystroglycan by POMGnT1 catalyzed GlcNAc modification. *Bioorg. Med. Chem.* **27**, 2822–2831 (2019).
2. Artigas, G., Hinou, H., Garcia-Martin, F., Gabius, H.-J. & Nishimura, S.-I. Synthetic Mucin-Like Glycopeptides as Versatile Tools to Measure Effects of Glycan Structure/Density/Position on the Interaction with Adhesion/Growth-Regulatory Galectins in Arrays. *Chem. Asian J.* **12**, 159–167 (2017).
3. Marcelo, F. *et al.* Delineating binding modes of Gal/GalNAc and structural elements of the molecular recognition of tumor-associated mucin glycopeptides by the human macrophage galactose-type lectin. *Chemistry* **20**, 16147–16155 (2014).
